# Recombinant HNP-1 Produced by Escherichia coli Triggers Bacterial Apoptosis and Exhibits Antibacterial Activity against Drug-Resistant Bacteria

**DOI:** 10.1128/spectrum.00860-21

**Published:** 2022-01-12

**Authors:** Qi Xie, Yin Wang, Mengmeng Zhang, Shujia Wu, Wei Wei, Weidi Xiao, Yihao Wang, Jinchao Zhao, Nan Liu, Yiguang Jin, Junzhu Wu, Ping Xu

**Affiliations:** a School of Basic Medical Science, Key Laboratory of Combinatorial Biosynthesis and Drug Discovery of Ministry of Education and Hubei Province Engineering and Technology Research Center for Fluorinated Pharmaceuticals, School of Pharmaceutical Sciences, Wuhan University, Wuhan, China; b State Key Laboratory of Proteomics, Beijing Proteome Research Center, National Center for Protein Sciences Beijing, Research Unit of Proteomics & Research and Development of New Drug of Chinese Academy of Medical Sciences, Institute of Lifeomics, Beijing, China; c Department of Neurology, Xinqiao Hospital, Army Medical University (Third Military Medical University), Chongqing, China; d Department of Pharmaceutical Sciences, Beijing Institute of Radiation Medicine, Beijing, China; e Anhui Medical University, Hefei, China; f School of Public Health, China Medical University, Shenyang, China; University of Calgary

**Keywords:** HNP-1, bacterial apoptosis, RecA, anti-MRSA, proteomics

## Abstract

Human neutrophil peptide-1 (HNP-1) is a promising antibiotic candidate, but its clinical applications have been hampered by challenges during mass production and an inadequate understanding of its bactericidal mechanisms. In this study, we demonstrated that Escherichia coli expressing full-length preproHNP-1 secretes a soluble form of HNP-1, which can be recovered from the total cell lysate after isopropyl thio-β-d-galactoside (IPTG) induction and ultrafiltration. Label-free quantitative proteomics and co-immunoprecipitation experiments revealed that HNP-1 induces cell apoptosis in bacteria by causing DNA and membrane damage. Notably, we found that HNP-1 disrupts the DNA damage response pathway by interfering with the binding of RecA to single-stranded DNA (ssDNA). Further experiments demonstrated that HNP-1 encapsulated in liposomes inhibits the growth of methicillin-resistant Staphylococcus aureus (MRSA) and meropenem-resistant Pseudomonas aeruginosa (MRPA). These results indicated that recombinant protein expression may be a simple and cost-effective solution to produce HNP-1 and that RecA inhibition via HNP-1 may serve as an alternative strategy to counteract antibiotic resistance.

**IMPORTANCE** Human neutrophil peptide-1 (HNP-1) is a promising antibiotic candidate, but its clinical application has been hampered by the difficulty of mass production and an inadequate understanding of its bactericidal mechanisms. In this study, we demonstrated that recombinant protein expression combined with ultrafiltration may be a simple and cost-effective solution to HNP-1 production. We further found that HNP-1 induces bacterial apoptosis and prevents its SOS repair pathway from binding to the RecA protein, which may be a new antibacterial mechanism. In addition, we showed that HNP-1 encapsulated in liposomes inhibits the growth of methicillin-resistant Staphylococcus aureus (MRSA) and meropenem-resistant Pseudomonas aeruginosa (MRPA). These results provide new insights into the production and antibacterial mechanism of HNP-1, both of which may promote its clinical application.

## INTRODUCTION

Antimicrobial resistance is a serious problem that endangers global public health globally. With the increase in antibiotic-resistant bacteria, there is an urgent need to develop novel antibiotics (1). Defensins, which are expressed by the host to eliminate invasive pathogens and enhance the immune response, are important candidates for the development of new antibiotics (2). The three types of mammalian defensins, α, β, and circular, have β-sheet structures stabilized by three disulfide bonds, but differ in the distribution and connection of six cysteine residues (3). As the main defensin in human neutrophils, α-defensin-1 (also called human neutrophil peptide-1 [HNP-1]) has been considered as a potential new antibacterial drug because of its wide spectrum of antibacterial activity (4). HNP-1 is first synthesized as preproHNP-1 by neutrophil precursor cells and then is proteolytically processed to from proHNP-1 in the endoplasmic reticulum, Golgi complex, and azurophilic granules before reaching full maturity (5). However, the amount of mature HNP-1 that can be obtained from human peripheral blood is extremely limited, and the cost for the chemical synthesis of this peptide is too expensive to allow for mass production. In addition, previous attempts to use a prokaryotic system to directly express mature HNP-1 have failed due to its cytotoxicity to the host cells. Various fusion protein methods have been used to reduce the toxicity of mature HNP-1 and increase its solubility, but the effects were not ideal (6, 7), possibly because HNP-1 has difficulty folding properly in a short period of time, and its toxic effects are difficult to neutralize. Thus, there is currently no suitable solution for the large-scale and low-cost production of mature HNP-1.

In addition to the low production of HNP-1, its clinical applications have been limited because its mechanism of killing resistant bacteria has not yet been elucidated. It is generally believed that HNP-1 causes bacterial death by penetrating the bacterial membrane due to its positive charge (8, 9). Some studies have also proposed that HNP-1 targets key intracellular processes (e.g., inhibition of protein, DNA, or cell wall synthesis) that lead to bacterial cell death without membrane disruption (10). However, antibacterial resistant bacteria differ from common Gram-positive or Gram-negative bacteria in that they resist antimicrobial agents through multiple mechanisms, such as repulsion or sequestration by bacterial surface structures, alterations in membrane charge or fluidity, or degradation and removal by efflux pumps (11). Based on the above mechanisms, recent studies have demonstrated that the SOS response is a cellular DNA repair mechanism that plays an essential role in antimicrobial-resistant bacteria in processes involving DNA damage-inducing stressors, such as those processes involving reactive oxygen species, detoxification, horizontal gene transfer, hypermutation states, biofilm formation, and the persistence and formation of small colony variants (12). In the presence of DNA damage, RecA is activated and binds to single-stranded DNA (ssDNA) to form a filamentous nucleoprotein. RecA then interacts with LexA and promotes the self-cleavage activity of LexA to induce SOS expression. Thus, inhibiting the SOS pathway or RecA activation may be a promising strategy to decrease bacterial resistance to antibiotics (13). Although there have been reports that HNP-1 can kill resistant bacteria, including Staphylococcus aureus and Pseudomonas aeruginosa (14, 15), it is unclear whether HNP-1 exerts a bactericidal effect through the SOS pathway.

In this study, we first constructed the preproHNP-1 plasmid to induce the expression and purification of HNP-1 in Escherichia coli and found that the mature peptide HNP-1 was successfully produced by this process. Next, we performed quantitative proteomics and co-immunoprecipitation experiments to explore the antibacterial mechanism of HNP-1. We demonstrated that RecA, as a key molecule to improve bacterial resistance, was a potential HNP-1-interacting protein. Then, we treated resistant bacteria, such as S. aureus and *Pneumococcus*, with HNP-1 to determine the role of HNP-1 in the cell death of resistant bacteria. Our results showed that HNP-1 promotes programmed bacterial death by binding to RecA protein, which may be a new bactericidal mechanism.

## RESULTS

### Isopropyl thio-β-d-galactoside (IPTG) induces the expression of preproHNP-1 and the production of HNP-1.

**FIG 1:**
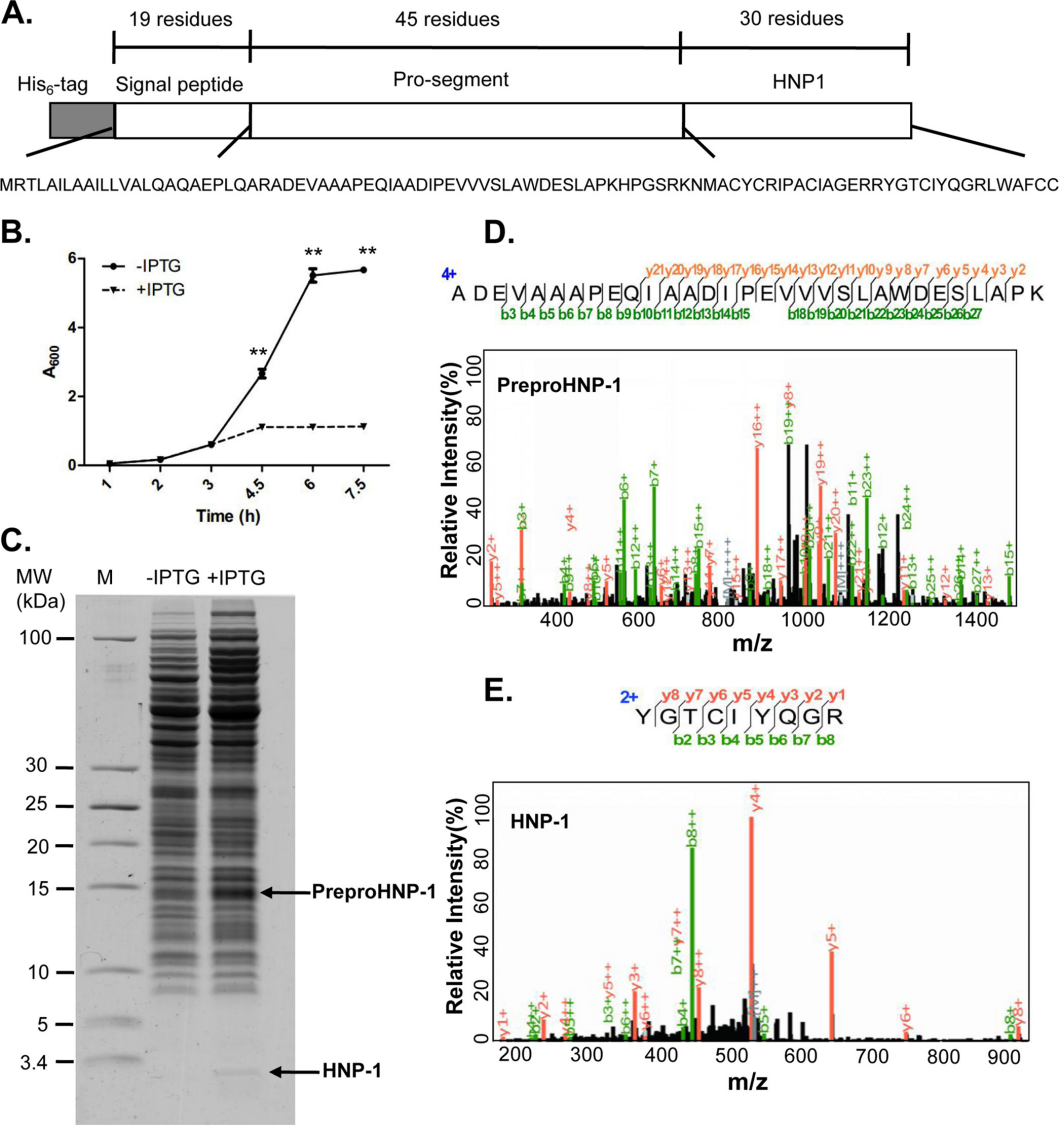
HNP-1 can be effectively produced in E. coli expressing preproHNP-1 after IPTG induction. (A) The sequence of preproHNP-1 tagged with six histidine residues, which was cloned into the pET-28a(+) vector. (B) Growth curves of E. coli strain XPX-1 containing pET-28a(+)-preproHNP-1 with (+IPTG) or without (−IPTG) 1 mM IPTG induction. Asterisks represent significant differences from the −IPTG group. (C) High-resolution Tris-Tricine gel analysis for the total cell lysates prepared from E. coli strain XPX-1 with (+IPTG) or without (−IPTG) 1 mM IPTG induction. PreproHNP-1 and HNP-1 are displayed as indicated. (D) Representative MS_2_ spectrum of peptide ADEVAAAPEQIAADIPEVVVSLAWDESLAPK from preproHNP-1. (E) Representative MS_2_ spectrum of peptide YGTCIYQGR from HNP-1. Data were analyzed using a two-tailed Student's *t* test and plotted as the mean ± SD for each condition. **, *P < *0.01.

An E. coli strain containing the preproHNP-1 sequence (XPX-1) was constructed specifically for this study (Fig. 1A). XPX-1 cells were grown to an approximate optical density (OD_600_) of 0.4 in 20 ml of Luria−Bertani medium containing kanamycin (50 μg/ml) at 37°C, and then IPTG was added at a final concentration of 1 mM to induce the expression of preproHNP-1. We observed that the growth of XPX-1 cells only reached an OD_600_ of approximately ∼1 (Fig. 1B), which was low compared with what one would expect from recombinant protein production (16, 17), suggesting that the expression of HNP-1 was suppressed.

To confirm our hypothesis, total cell lysate (TCL) samples prepared from XPX-1 cells with and without IPTG induction were loaded onto a high-resolution Tris-Tricine gel. We found that TCL with IPTG treatment exhibited bands at 15 kDa and 3 kDa (Fig. 1C), which corresponded to the theoretical molecular weights of preproHNP-1 (14.8 kDa) and HNP-1 (3.4 kDa), respectively (18). For further confirmation, the gel bands were excised, digested, and analyzed using mass spectrometry (MS). We identified the tryptic peptide ADEVAAAPEQIAADIPEVVVSLAWDESLAPK for preproHNP-1 in four of the MS spectra from the gel bands of the IPTG-treatment TCL (Fig. 1D). The sequencing coverage reached 53.60% for the full-length preproHNP-1 and 62.20% for its sequenceable portion (Table S2 in the supplemental material). In addition, we identified two tryptic peptides with a high-quality MS_2_ spectrum for HNP-1 (Fig. 1E) in the 3-kDa gel band. The sequencing coverage was 60.00% for full-length HNP-1 and 100% for its sequenceable portion. We further compared the retention time, chromatography peaks, charge state distribution, and *m/z* value from the 3.4-kDa band with those of a commercially purchased HNP-1 standard (Sigma-Aldrich, St. Louis, MO, USA) and found that they were very similar (Fig. S1A and B). These results suggested that IPTG induced the production of HNP-1 from preproHNP-1.

### HNP-1, but not preproHNP-1, arrests the growth of E. coli.

**FIG 2:**
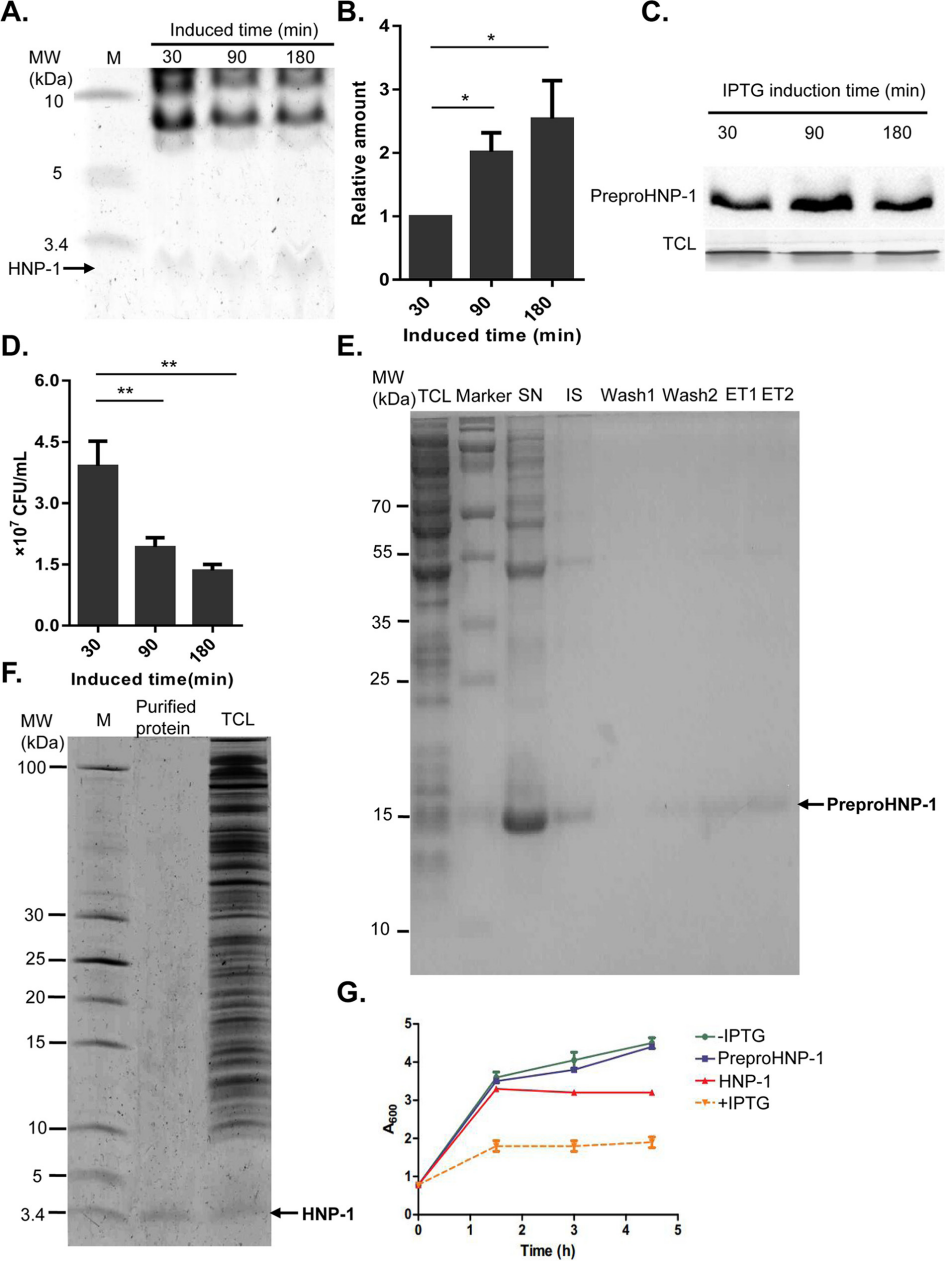
Increased antibacterial activity of the endogenous recombinant HNP-1. (A) The production of HNP-1 after IPTG induction. The induction time points of IPTG are labeled as indicated. (B) Quantitative analysis of the HNP-1 band in Fig. 2A by mass spectrometry. (C) The production of preproHNP-1 by IPTG induction analyzed by Western blot. The induction time points of IPTG are labeled as indicated. (D) Bacterial colony count after IPTG induction. The induction time points are labeled as indicated. The number of viable cells were counted after growing for 16 h on plates containing Luria-Bertani broth media. (E) PreproHNP-1 protein purification. TCL: total cell lysate; SN: supernatant; IS: insoluble protein; ET: elution. (F) Tris-Tricine gel analysis of the purified HNP-1. The TCL was saved as control. (G) Comparison of the antibacterial activity of the purified HNP-1. The growth curve of E. coli strain XPX-1 with 1 mM IPTG induction as a positive control and XPX-1 without IPTG induction as a negative control. PreproHNP-1 and HNP-1 represent treatment with the purified recombinant preproHNP-1 (20 μg/ml) and HNP-1 (20 μg/ml), respectively. Data were analyzed using a two-tailed Student's *t* test and are plotted as the mean ± SD for each condition. *, *P < *0.05 and **, *P < *0.01.

We wondered whether the observed growth arrest (Fig. 1B) was caused by the expression of preproHNP-1 or the production of HNP-1. By separating the 3.4 kDa-sized band from a Coomassie blue-stained Tris-Tricine gel, we showed that the intensity of the HNP-1 band gradually increased with induction time (Fig. 2A) and that the amount of HNP-1 produced after 3 h was 2.5 times greater than that produced after 0.5 h (Fig. 2B). However, the expression of preproHNP-1 was the highest at 1.5 h, and then its expression level decreased (Fig. 2C), suggesting that HNP-1 was indeed the product of IPTG-induced preproHNP-1 expression. More interestingly, we found that the number of viable bacteria significantly decreased with induction time (Fig. 2D). The negative correlation between the amount of HNP-1 produced and the number of viable bacteria suggested that HNP-1 exhibits antibacterial activity.

To confirm the antibacterial activity of HNP-1, we purified preproHNP-1 and HNP-1 from the TCL after IPTG induction. Specifically, preproHNP-1 was enriched with six histidine tags followed by Tris-Tricine gel separation (Fig. 2E), whereas HNP-1 was isolated under native conditions via ultrafiltration. The high purity of HNP-1 was verified by Tris-Tricine gel analysis (Fig. 2F). We compared the effects of preproHNP-1 on E. coli with those of HNP-1. The constructed growth curves showed that E. coli cultured in growth medium containing purified preproHNP-1 did not differ from those cultured in regular growth medium without IPTG induction. However, growth medium containing purified HNP-1 inhibited the growth of E. coli in much the same way as regular medium with IPTG treatment, although to a lesser degree of approximately 50% (Fig. 2G). The result strongly suggested that purified HNP-1, not preproHNP-1, has antibacterial activity.

**FIG 3:**
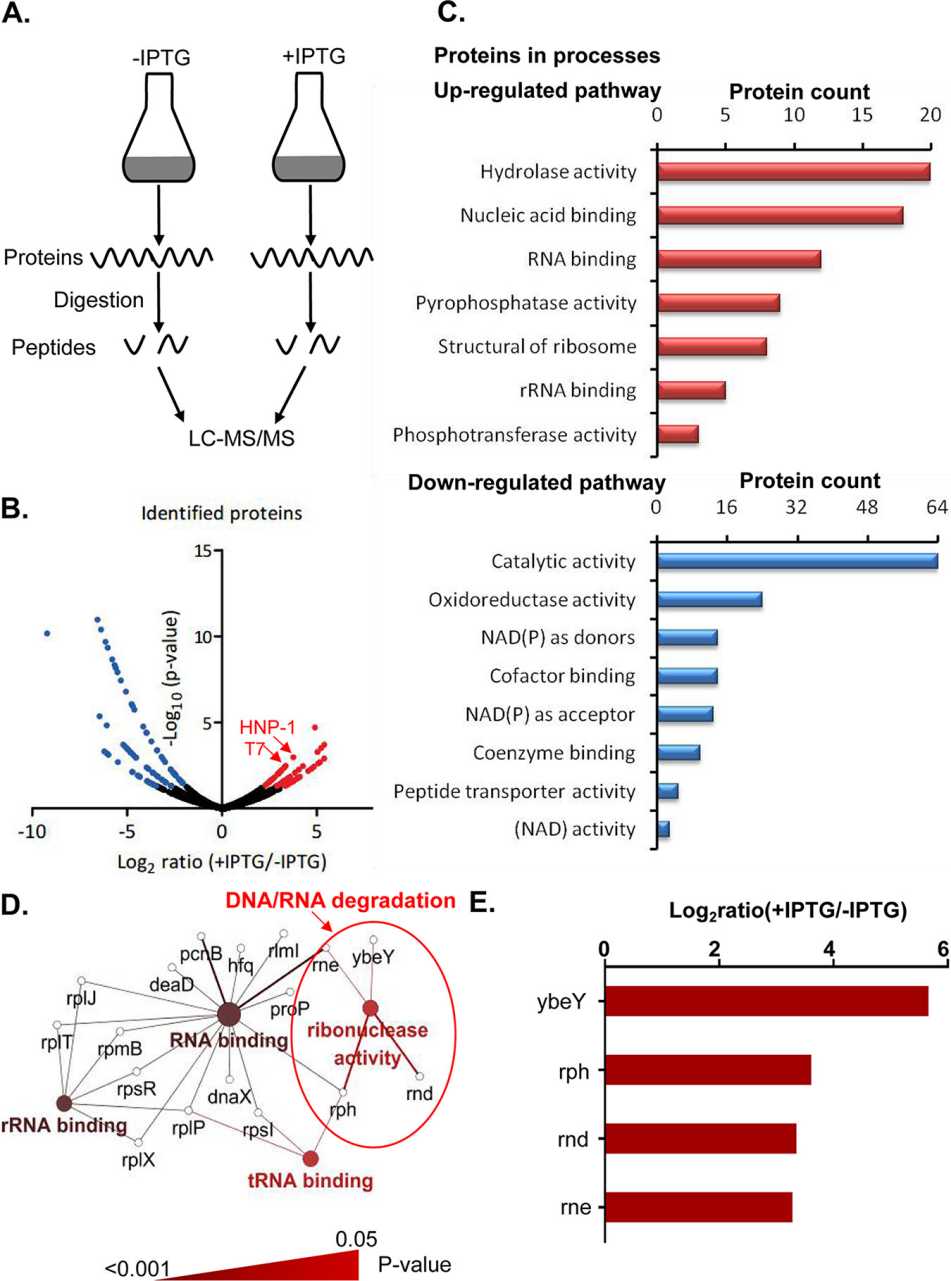
Quantitative proteomics reveals the activation of the apoptosis pathway after the expression of HNP-1 in E. coli. (A) Flowchart of the investigation of the antibacterial mechanism of HNP-1 through label-free quantitative proteomics. (B) Volcano plot representation of the quantified proteins. The red spots indicate the proteins that were upregulated, and the blue spots indicate the proteins that were downregulated after the expression of HNP-1. (C) Gene Ontology categories of the biological processes of the differentially expressed proteins. (D) KEGG pathway analysis of the upregulated proteins after the expression of HNP-1. Large nodes represent pathways within core regulatory networks. Enzymes are represented by small nodes. The pathways with *P < *0.05 and cluster protein number ≥ 3 are displayed. (E) The expression of endonucleases after IPTG induction.

To understand how HNP-1 inhibits E. coli, we performed a label-free quantitative proteomics analysis on XPX-1 cells with and without IPTG induction (Fig. 3A). In total, we identified 1,559 proteins, of which 1,431 proteins were found in both XPX-1 cell groups. By comparing the intensities of each protein between the two XPX-1 cell groups, we screened for differentially expressed proteins using the significant B algorithm that was provided by the Perseus software (19). Setting the threshold at *P < *0.05 resulted in 152 differentially expressed proteins (Fig. 3B), of which 66 proteins were upregulated proteins and 86 were downregulated proteins (Table S3 in the supplemental material). We noted that both HNP-1 and IPTG-induced T7 RNA polymerase were significantly upregulated, indicating the high reliability of our quantitative proteomics results. Gene Ontology classification (20) suggested that the upregulated proteins were predominantly related to hydrolase activity and nucleic acid binding, and the downregulated proteins were associated with oxidoreductase activity (Fig. 3C). The clustering results showed that endonucleases were upregulated (Fig. 3D and E). In particular, the apoptotic-like signals characterized by ybeY and other endonucleases were activated (21), suggesting that HNP-1 can also cause bacterial death by inducing bacterial apoptosis.

### HNP-1 triggers bacterial apoptosis in E. coli and drug-resistant bacteria.

**FIG 4:**
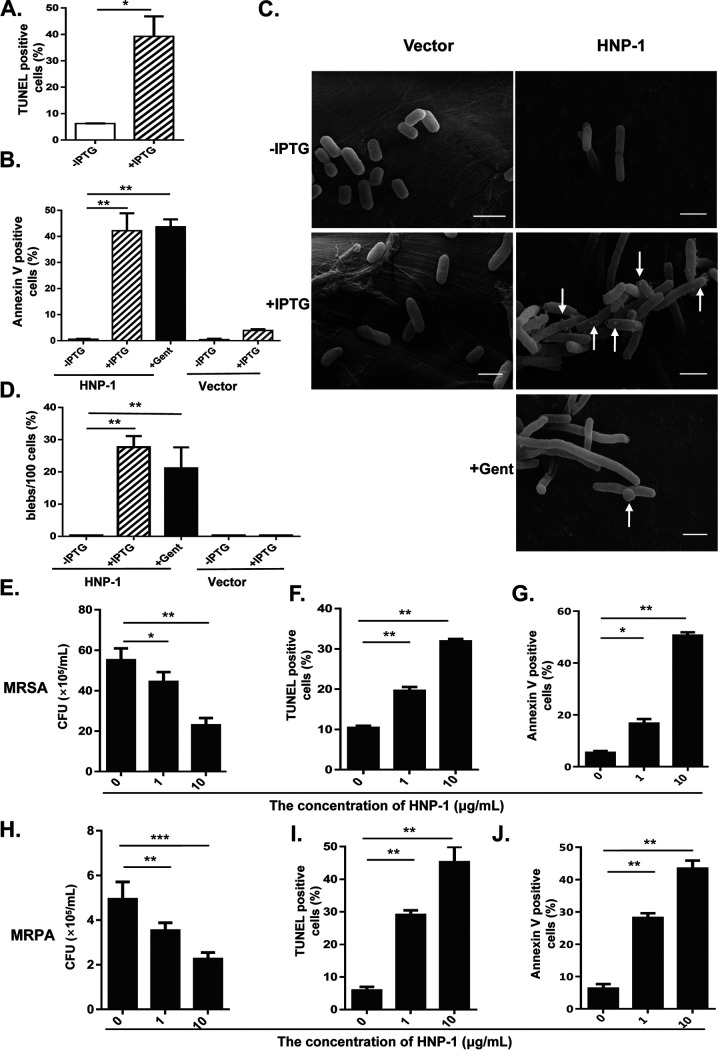
HNP-1 induced apoptosis in E. coli, MRSA, and MRPA. (A) The percentage of TUNEL-positive E. coli at 3 h after IPTG induction. (B) The percentage of Annexin V-labeled E. coli at 3 h after IPTG induction. The E. coli strain XPX-2 (labeled as Vector) was saved as a negative control. Gentamicin (Gent, 10 μg/ml) was used as a positive control because it is a known drug that induces bacterial apoptosis. (C) Scanning electron micrograph of the morphology of the E. coli strain. The white arrows indicate blebs after HNP-1 expression was induced with 1 mM IPTG; scale bar, 2 μm. (D) The percentage of blebs/per hundred cells 3 h after IPTG induction. (E) Effects of different concentrations of synthetic HNP-1 on the growth of MRSA. (F) The percentage of TUNEL-positive MRSA at 3 h after HNP-1 treatment. (G) The percent of Annexin V-labeled MRSA at 3 h after HNP-1 treatment. (H) Effects of different concentrations of recombinant HNP-1 on the growth of MRPA. (I) The percentage of TUNEL-positive MRPA 3 h after HNP-1 treatment. (J) The percent Annexin V-labeled MRPA 3 h after HNP-1 treatment. Data were analyzed using a two-tailed Student's *t* test and are plotted as the mean ± SD for each condition. *, *P < *0.05; **, *P < *0.01; and ***, *P < *0.001.

Bacterial apoptosis is characterized by membrane depolarization and DNA fragmentation, both of which are also hallmarks of eukaryotic mitochondrial apoptosis. We speculated that the expression of HNP-1 might trigger bacterial apoptosis via a similar mechanism. To test this hypothesis, we measured the levels of DNA fragmentation, a hallmark of apoptosis, in XPX-1 cells treated with HNP-1 using a TUNEL (terminal deoxynucleotidyl transferase (TdT)-mediated dUTP-biotin nick end labeling) assay. As expected, we found that HNP-1 efficiently induced DNA fragmentation (Fig. 4A).

During apoptosis, the phosphatidylserine (PS) typically facing the cytoplasm is exposed on the outside of the cytoplasmic membrane. The relocalization of PS to the outer cell surface is the basis for Annexin V staining (22). Therefore, to further confirm that HNP-1 caused apoptosis, we also stained XPX-1 cells with Annexin V and showed that the expression of HNP-1 induced bacterial apoptosis after IPTG induction (Fig. 4B). However, there was no visible indicators of apoptosis in control E. coli even after treatment with IPTG (1 mM).

A physiological characteristic that is associated with apoptosis is the formation of membrane protrusions called blebs (23, 24). We inspected the membrane structure of XPX-1 cells during HNP-1 mediated apoptosis using scanning electron microscopy (SEM) and found extensive blebbing (Fig. 4C and D). In addition, control E. coli cell had no noticeable morphological changes, even with IPTG treatment. Moreover, IPTG induction resulted in larger, almost filamentous cells compared to the vector control, and gentamicin (Gent) conditions showed similar filamentation (Fig. 4C). Filamentation is the anomalous growth of certain bacteria, such as E. coli, in which cells continue to elongate but do not divide. The cells that result from elongation without division have multiple chromosome copies (25). Antimicrobial peptide (AMP)-induced filamentation has also been described for HNP-1, HNP-2, HNP-5, indolicidin, and the proline-arginine-rich antimicrobial peptide PR-39 (26, 27). We thus wondered whether HNP-1 could promote apoptosis in drug-resistant bacteria. Therefore, methicillin-resistant Staphylococcus aureus (MRSA) and meropenem- resistant Pseudomonas aeruginosa (MRPA) were selected for further validation. Our results showed that bacterial growth was significantly reduced at an HNP-1 concentration of 10 μg/ml (Fig. 4E and H). HNP-1 treatment yielded a high percentage of TUNEL-positive cells (Fig. 4F and I). We also observed significant numbers of Annexin V-positive cells at an HNP-1 concentration of 10 μg/ml (Fig. 4G and J). These data suggested that HNP-1 treatment induces cell apoptosis in MRSA and MRPA.

### HNP-1 binds RecA to inhibit its activity.

**FIG 5:**
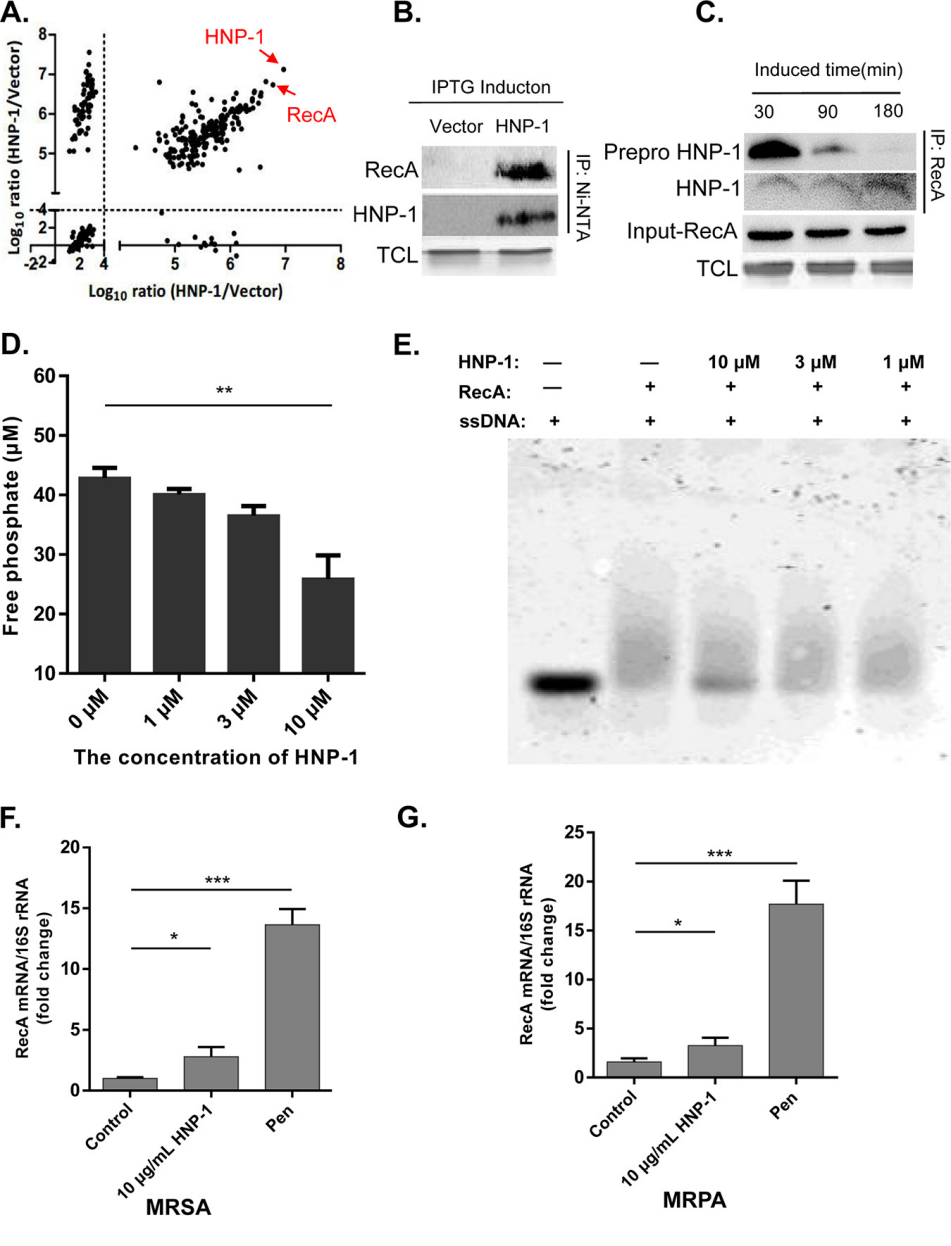
HNP-1, but not full length preproHNP-1, interacts with RecA. (A) Global distribution of HNP-1-interacting proteins in biological replicate samples. The proteins with log_10_ intensity ratio >4 were selected for further analysis. HNP-1 itself and heavily enriched RecA are labeled with red arrows. (B) The His_6_ tagged HNP-1 interacted with RecA. HNP-1 was enriched through a 6x His-tag for purification with Ni-NTA beads and probed with RecA antibody. (C) HNP-1, but not full length 6x His-tagged preproHNP-1, interacted with RecA. Cells expressing HNP-1 were collected at different time points as indicated. RecA was immunoprecipitated with an anti-RecA antibody, followed by Western blotting using an anti-HNP-1 antibody. (D) HNP-1 inhibition of ssDNA-stimulated RecA ATPase activity. ATPase activity was measured by monitoring the release of inorganic phosphate using a malachite green phosphate detection assay. (E) HNP-1 inhibited RecA-ssDNA binding. RecA-bound ssDNA and free ssDNA were resolved using an agarose gel. (F) Quantification of RecA mRNA in MRSA by real-time RT-PCR. (G) Quantification of RecA in MRPA mRNA by real-time RT-PCR. Data were analyzed using a two-tailed Student's *t* test and are plotted as the mean ± SD for each condition. *, *P < *0.05 and ***, *P < *0.001.

To determine the underlying mechanism of HNP-1 mediated cell apoptosis, we screened for proteins that interact with HNP-1 by co-immunoprecipitation followed by LC-MS/MS. Our results showed that preproHNP-1 and histidine tags were both enriched (Fig. S2 in the supplemental material), suggesting that the enrichment of HNP-1 and its interacting proteins was successful. To identify the HNP-1-interacting proteins more accurately, we eliminated potential contaminants during purification and analyzed samples in duplicate. After filtering (log_10_ intensity of HNP-1/control > 4), we identified 186 potential interacting proteins, including preproHNP-1 itself (Fig. 5A and Table S4). Among these potential interacting proteins, RecA ranked at the top with a sequencing coverage of over 47% (Table S5). This result was further confirmed by Western blot analysis (Fig. 5B).

RecA is one of the most studied SOS response inducers of DNA repair in bacteria (21). However, it is not clear how preproHNP-1 or HNP-1 itself regulates bacterial apoptosis through RecA. To clarify whether HNP-1 itself or another portion of preproHNP-1 interacted with RecA, we performed a series of co-immunoprecipitation experiments with an anti-RecA antibody and XPX-1 proteins collected at different time points after IPTG induction. We noted that preproHNP-1 accumulation always preceded HNP-1 production. Co-precipitated preproHNP-1 decreased with time, but co-precipitated HNP-1 increased at nearly the same rate as RecA (Fig. 5C). This result suggested that RecA interacts with HNP-1 instead of preproHNP-1.

As a DNA-dependent ATPase, RecA protein hydrolyzes ATP when bound to ssDNA, and its rate of ATP hydrolysis generally correlates to the amount bound. Therefore, we assessed the ability of recombinant HNP-1 to inhibit RecA ATPase activity in the presence of ssDNA as described previously (28). In the absence of inhibitor, we observed a dramatic increase in the amount of hydrolyzed ATP. However, the addition of HNP-1 to the RecA-ssDNA mixture slowed ATP hydrolysis (Fig. 5D). Moreover, HNP-1 inhibited ssDNA-binding activity in the micromolar range (Fig. 5E), suggesting that HNP-1 inhibits RecA ATPase activity and DNA-binding ability. As a key molecule in the SOS response, RecA is activated under antibiotic pressure, which can result in drug resistance, such as in MRSA and MRPA. In addition, we found that the expression of RecA increased significantly after MRSA and MRPA were treated with penicillin, while the expression of RecA was almost unchanged after HNP-1 treatment (Fig. 5F and G), further confirming that HNP-1 kills resistant bacteria by inhibiting the expression of RecA, which, in turn, inhibits the DNA repair functions of bacteria and lowers their chance of survival (Fig. S3 in the supplemental material).

### Liposomal HNP-1 attenuates lung injury in rats with MRSA-induced pneumonia.

**FIG 6:**
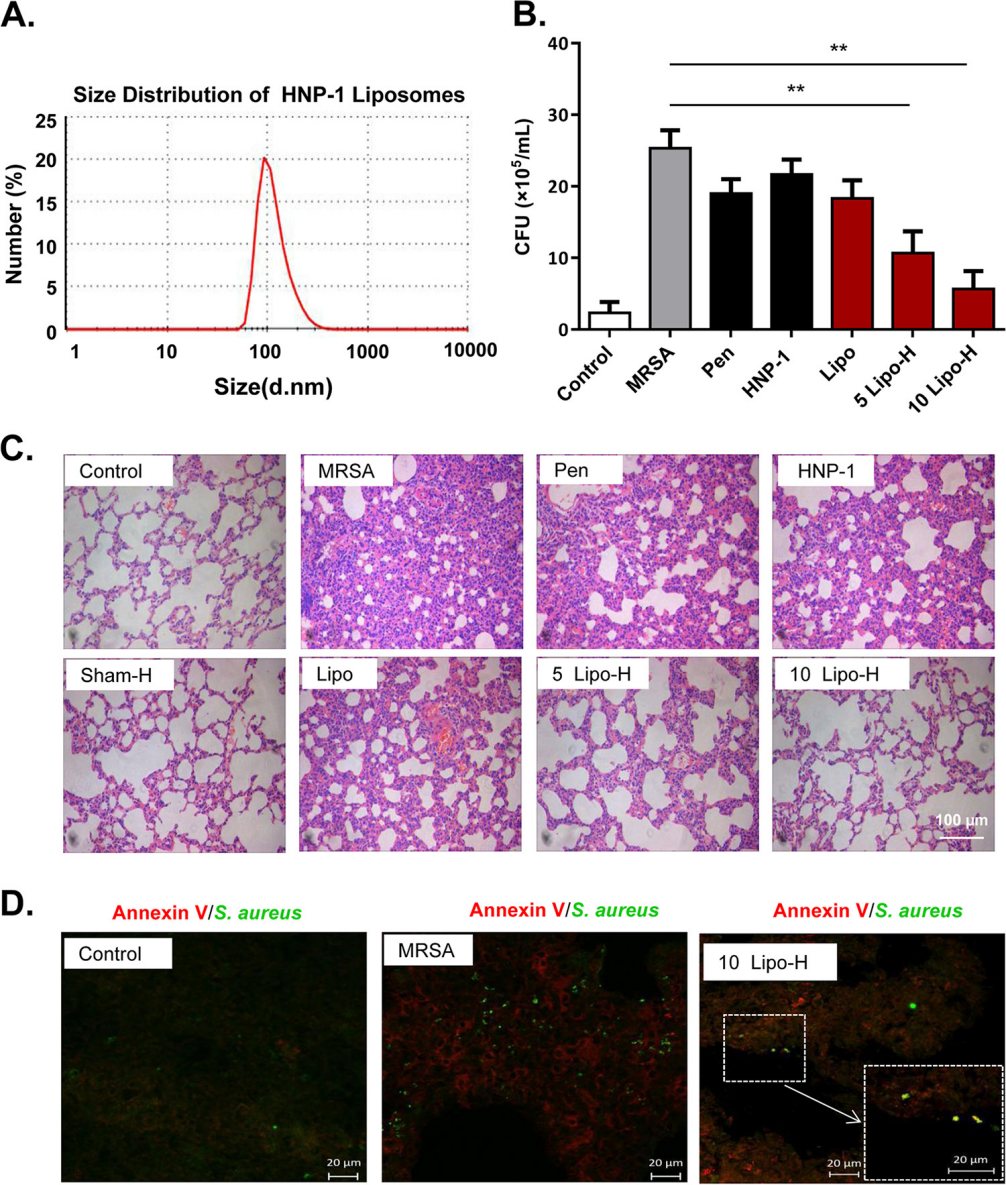
HNP-1 liposomes attenuated MRSA-induced lung injury in rats. (A) Size distribution of HNP-1 liposomes based on the results from a Zetasizer Nano ZS (Malvern, UK). (B) Comparison of the bacterial counts in the different treatment groups. The number of MRSA isolated from lung lavage fluid were counted on the agar-containing culture plates followed by culturing for 36 h at 37°C. The rats were divided into seven groups: 1) healthy control rats (Control); 2) pneumonic rat models induced by MRSA; 3) 12 μg/ml penicillin treatment as the quality control to ensure the strains are resistant to penicillin (Pen); 4) pneumonic rats treated with 0.1 ml 20 μg/ml HNP-1 solutions (2 μg of HNP-1); 5) pneumonic model rat treated with blank liposomes (Lipo); 6) pneumonic model rats treated with 0.1 ml of 5 μg/ml of liposome-coated HNP-1 (0.5 μg of Lipo-H); and 7) pneumonic model rats treated with 0.1 ml 10 μg/ml liposome-coated HNP-1 (1 μg of Lipo-H). (C) Pathological evaluation via H&E staining in pneumonic model rats. (D) Immunofluorescent staining for Annexin V (red) and S. aureus (green) in pneumonic model rats. Merged images (yellow) indicating of S. aureus apoptosis. Data were analyzed using a two-tailed Student's *t* test and are plotted as the mean ± SD for each condition. *n* = 3; *, *P < *0.05; and **, *P < *0.01.

Previous studies have shown that the plasma composition can significantly affect the antibacterial activity of HNP-1 (29). In addition, our findings suggested that endogenous HNP-1 exhibits antibacterial activity more effectively than exogenous HNP-1. More importantly, we have previously shown that encapsulation of drugs in liposomes can increase their potency against MRSA-induced inflammatory diseases (30). For these reasons, we wanted to evaluate the antibacterial activity of liposomal HNP-1 *in vivo* and *in vitro*. Therefore, we encapsulated HNP-1 in liposomes so that the peptide was protected and could better diffuse through bacterial membranes. Laser diffraction analysis showed that liposomal HNP-1 had a mean particle size of 116.3 nm (Fig. 6A).

The *in vitro* antibacterial activity results showed that both HNP-1 and liposomal HNP-1 inhibited the growth of MRSA, and the antibacterial activity was not significantly different between these two treatments (Fig. S4). We sprayed MRSA-induced pneumonic rats with liposomal HNP-1 and afterward checked their lung lavage fluids for bacterial clearance. Our results showed that pneumonic rats with liposomal HNP-1 treatment had significantly fewer CFU (CFU) than those without treatment (**, *P < *0.01). Interestingly, the effects were dose-dependent, and liposomal HNP-1 displayed bacterial clearance effects at dosages as low as 1 μg/day/rat. However, neither HNP-1 nor penicillin treatment had any effect on bacterial clearance effects (Fig. 6B), suggesting that only liposomal HNP-1 was effective MRSA *in vivo*.

Because of the important roles of HNP-1 in innate immunity, we examined the immune cells of MRSA-induced pneumonic rats that had undergone liposomal HNP-1 treatment. Both the leukocyte and neutrophil levels were significantly decreased as compared with untreated or pneumonic rats that had undergone HNP-1 treatment (Fig. S5A and B in the supplemental material). In addition, the level of the proinflammatory cytokine interleukin-6 (IL-6) decreased (Fig. S5C), and the level of interferon-γ (IFN-γ) increased (Fig. S5D). These results suggested that the bactericidal effects of HNP-1 are achieved through either bacterial apoptosis or immune regulation. H&E-stained sections of the terminal airways and interstitium showed that there was no obvious eukaryotic cell damage after intraperitoneal injection of a low dosage (2 μg/day) of HNP-1 to healthy rats, whereas MRSA-induced pneumonic rats displayed exudation, inflammation, and severe hemorrhage (Fig. 6C). Although HNP-1 or penicillin treatment alleviated these symptoms to a certain degree, only liposomal HNP-1 was able to alleviate all of the symptoms, as well as lung injury. Annexin V staining of histological sections further confirmed that bacterial apoptosis occurred in MRSA in the rats treated with liposomal HNP-1 (Fig. 6D).

## DISCUSSION

AMPs are nature’s most powerful weapon against microbes, including drug-resistant bacteria. However, the research and development of AMPs have been hampered by their low abundance in natural sources, as well as the difficulty of mass production. In this study, we demonstrated the possibility of manufacturing HNP-1 by expressing a full-length preproHNP-1 in E. coli. Quantitative analysis showed that the concentration of preproHNP-1 was 0.6 μg/μl, and the concentration of HNP-1 was 0.56 μg/ml (Fig. S6 in the supplemental material). In addition, Tris-Tricine gel analysis showed that the molecular weight of HNP-1 was significantly less than that of other endogenous proteins found in E. coli. Proteomics data further confirmed that HNP-1 was the major protein expressed at a molecular weight below 10 kDa after IPTG induction (Table S6). Given the small molecular weight, we decided to use ultrafiltration as the method to isolate HNP-1 after production. Our results showed that ultrafiltration can indeed generate relatively pure HNP-1 with functional antimicrobial activity.

One of the most important but least understood aspects of AMPs, is their antibacterial mechanisms. The current consensus is that AMPs exert broad-spectrum antimicrobial activity via membrane permeabilization (31, 32). However, there has been speculation that transmembrane pore formation is not the only bactericidal mechanism. As a result, various other models have been proposed, such as septum formation in the cytoplasmic membrane, inhibition of cell-wall synthesis (33), and bacterial apoptosis (22). In this study, we showed that HNP-1 can indeed disrupt the regulatory network of apoptosis pathways. Moreover, our SEM analysis and Annexin V and TUNEL staining assays showed that both the bacterial membrane and DNA were destroyed after HNP-1 treatment. These results demonstrated that HNP-1 triggered cell apoptosis in bacteria. Although there have been reports that HNP-1 elicits apoptosis in eukaryotic cells and neutrophils (34, 35), to our knowledge, we are the first to show that HNP-1 induces apoptosis in bacteria.

In addition, our work provides detailed mechanistic insights into a bactericidal mechanism that is potentiated by disrupting RecA and disabling SOS responses (36). Several inhibitors of RecA activity have been discovered in recent studies, including metal cations, small molecules, and chemically modified peptides (37–39). It is generally believed that the purified HNP-1 protein inhibits RecA activity *in vitro* in the 1–20 μm range *in vitro* (40, 41). Our results showed that HNP-1 inhibits RecA activity by inhibiting its ability to bind to ssDNA at a concentration of 10 μm, suggesting that HNP-1 is a potential RecA inhibitor for drug-resistant bacterial therapy. Other human defensins, such as beta-defensins, have been shown to have anti-staphylococcal properties and are associated with persistent MRSA colonization (42, 43). However, its interaction with RecA to inhibit the SOS activity of resistant strains has not been reported before. Our study found that HNP-1 can inhibit the expression of RecA in MRSA and MRPA, which would be a key mechanism against drug-resistant bacteria.

Unlike antibiotics, which are chemically stable, the clinical application of AMPs has been limited by their susceptibility to proteolysis and low antibacterial activity under physiological conditions (44). These findings are consistent with what we have observed in our studies. To overcome these limitations, we encapsulated HNP-1 in liposomes to provide better protection and enhance its antibacterial activity (30, 45). Unsurprisingly, liposomal HNP-1 was extremely effective, as it inhibited bacterial growth and reduced the inflammatory response and hemorrhage in the lungs of MRSA-infected rats *in vivo*. These results are encouraging, because they suggest the possibility of using liposomes for drug delivery to treat drug-resistant bacterial infections. Although resistance against defensins evolves readily in *in vitro* systems, this does not seem to be the case *in vivo* (46). In addition, antibiotic-resistant bacteria show a high frequency of collateral sensitivity to AMPs, whereas cross-resistance is relatively rare (47), which suggests that liposome-coated HNP-1 may be used in combination with certain antibiotics to kill resistant bacteria more efficiently.

## MATERIALS AND METHODS

### Recombinant strain construction and growth rate assessment.

The E. coli strains and plasmids used in this study are described in Table S1 in the supplemental material. The protocols are provided in the Supplementary Materials and Methods.

### Tris-Tricine gel analysis.

Tris-Tricine gel analysis was performed as described previously (18). The gel was stained with Coomassie brilliant blue G-250. After destaining, the gel was scanned using a scanjet image system (HP Scanjet G4050, China) and analyzed using Scion Image (http://rsb.info.nih.gov/nihimage/).

### HNP-1 purification.

PreproHNP-1 was purified from E. coli under denaturing conditions (48), as described in the Supplementary Materials and Methods. To purify HNP-1, 80 ml of XPX-1 cells harvested 3 h after induction with 1 mM IPTG was suspended in 500 μl of cold PBS (pH 7.2). The mixture was disrupted using a Soniprep Sonicator (Scientz, Ningbo, China) at the power of 25% for 25 cycles. The resultant TCL was centrifuged at 10,000 g for 25 min. The supernatant was loaded into an Amicon Ultra-0.5 ml centrifugal concentration unit (3 kDa MWCO, Millipore, Burlington, MA, USA) and centrifuged at 5,000 g for 10 min to recover the filtered solution. This step was repeated three times. All filtered solutions were pooled for quality checks and then diluted to a concentration of 20 μg/ml before use in antimicrobial activity assays.

### Proteomics analysis.

The extracted proteins were separated by Tricine-SDS-PAGE, and then gels were sliced into different gel pieces based on molecular weight markers and digested with 10 ng/μl trypsin (Meizhiyuan Scientific, Beijing, China) at 37°C overnight. The tryptic peptides were extracted with extraction buffer (45% acetonitrile with 5% formic acid), followed by acetonitrile, and then ultimately dried using a vacuum dryer (Labconco CentriVap, Kansas City, MO, USA). Before LC-MS/MS (Thermo Fisher Scientific, Waltham, MA, USA) analysis, the digested peptides were resuspended in loading buffer (1% acetonitrile and 1% formic acid in double distilled water). MS/MS raw files were processed using MaxQuant. Details are provided in the Supplementary Materials and Methods.

### Validation of the HNP-1 sequence and top-down proteomics.

HNP-1 was extracted from the gel using the method of Cohen and Chait (49). Briefly, the TCL prepared from XPX-1 cells 3 h after induction was loaded onto a 12% Tris-Tricine gel. The gel piece at approximately 3.4 kDa was excised and chopped into 1-mm^3^ cubes. After destaining, the cubes were placed into a mortar and crushed into a powder using a pestle and liquid nitrogen. The gel powder was dehydrated, and proteins were extracted with buffer containing 5% formic acid and 50% acetonitrile. The top-down proteomics parameters are detailed in Supplementary Materials and Methods.

### Preparation of liposomal HNP-1 and pneumonic rats.

HNP-1 liposomes were prepared using the method of Kim et al. (50). Rats were housed in a pathogen-free barrier facility accredited by the Association for Assessment and Accreditation of Laboratory Animal Care. Details are provided in the Supplementary Materials and Methods.

### Preparation of pneumonic rats and medicine administration.

MRSA and MRPA were isolated from the Affiliated Hospital of Xuzhou Medical University (Jiangsu, China) and obtained from Professor Shengqi Wang (Xuzhou Medical University, Jiangsu, China). Bacterial pneumonia rats were prepared by spraying MRSA suspensions into the lungs via the trachea with a long soft plastic tube, as described in our previous research (30). The rats were inoculated using tracheal intubation with 0.2 ml of 10^8^ CFU/ml MRSA suspensions. After 8 h, the rats were given medication. Twenty-one rats were divided equally into seven groups: i) healthy control rats; ii) pneumonic model rats treated with saline; iii) pneumonic model rats treated with blank liposomes; iv) pneumonic model rats treated with 0.1 ml of a 20 μg/ml HNP-1 solution; v) pneumonic model rats treated with 0.1 ml of 5 μg/ml HNP-1 liposomes; vi) pneumonic model rats treated with 0.1 ml of 10 μg/ml HNP-1 liposomes; and vii) pneumonic model rats treated with a 12 μg/ml sodium penicillin solution as the quality control to ensure that the strains were resistant to penicillin. All of the medicines were sprayed into the rat lungs through the trachea using an intratracheal MicroSprayer Aerosolizer (IA-1B; PennCentury Inc., Wyndmoor, PA, USA) once daily for 3 days without anesthesia.

### ATPase assay.

The rate of RecA-mediated ATP hydrolysis was measured using an ATPase activity assay (Sigma, St. Louis, MO, USA), as described previously (37) with slight modifications. Poly-dT was synthesized as ssDNA to avoid complexity from natural DNA (28). The ssDNA-dependent ATPase reaction was initiated by adding HNP-1 to a mixture composed of 1 μM RecA (New England Biolabs, Ipswich, MA, USA), 6 μM ssDNA, 1 mM ATP (Sigma, St. Louis, MO, USA), 40 mM Tris, 80 mM NaCl, 8 mM MgAc_2_, and 1 mM EDTA (pH 7.5). The absorbance at 630 nm due to green complexes formed between malachite green and free phosphate was measured.

### ssDNA oligonucleotide-binding assay.

RecA (15 μM), fluorescein-labeled oligonucleotide FAM43 (1 μM) (37), and the indicated amounts of HNP-1 were incubated in a binding buffer (10 mM MgCl_2_, 70 mM Tris-HCl, pH 7.6) for 30 min at 37°C. Bound and unbound RecA were resolved using a 2.0% agarose gel. Fluorescence emission was captured using a Typhoon 9410 Gel and Blot Imager (Amersham Biosciences, USA).

### General methodology.

The methodologies of proteomics analysis, SEM analysis, TUNEL staining, Annexin V staining and Western blot analysis methodologies are detailed in the Supplementary Materials and Methods.

### HNP-1 quantitative densitometric analysis.

The extracted XPX-1 protein (∼200 μg) was separated by Tricine-SDS-PAGE. The gel was stained with Coomassie brilliant blue G-250 and was scanned using a scanjet image system. The total protein band densities, and HNP-1 band densities were quantified by densitometric analysis using Scion image software.

### Statistical analysis.

Data are expressed as the mean ± SD and were analyzed using GraphPad Prism version 5 software. Statistical comparisons were evaluated by *t* test, and differences were considered statistically significant at *P < *0.05.
