# Supplementary material for: Recombinant HNP-1 Produced by Escherichia coli Triggers Bacterial Apoptosis and Exhibits Antibacterial Activity against Drug-Resistant Bacteria
Source: Microbiol Spectr. 2022 Jan 12;10(1):e00860-21. doi: 10.1128/spectrum.00860-21 (PMC8754131; doi:10.1128/spectrum.00860-21)
Supplement: SUPPLEMENTAL FILE 1 — Supplemental material. Download SPECTRUM00860-21_Supp_1_seq2.pdf, PDF file, 0.7 MB [file spectrum00860-21_supp_1_seq2.pdf]

Recombinant HNP-1 produced by *Escherichia coli* triggers bacterial apoptosis and exhibits anti-bacterial activity against drug-resistant bacteria

Qi Xie, Yin Wang, Mengmeng Zhang, Shujia Wu, Wei Wei, Weidi Xiao, Yihao Wang, Jinchao Zhao, Nan Liu, Yiguang Jin, Junzhu Wu, Ping Xu

Supplementary Materials and Methods

***E. coli* strains and plasmids.** *E. coli* strain BL21 (DE3), *E. coli* strain DH5α and vector pET-28a(+) were stored at −80 °C in our laboratory. The DNA sequence of preproHNP-1 and flanked with *EcoR* I and *Xho* I was generated from synthetic oligonucleotides according to a reference sequence (NM_004084.3). The products were ligated into the pET-28a(+) vector encoding a fragment with six histidine (His_6) at the N-terminus.

The resulting vectors were transformed into the *E. coli* DH5α cells and after isolation and purification, they were subjected to the DNA sequence analysis. After verifying the sequence, the vectors were transformed into *E. coli* BL21 (DE3) competent cells to obtain XPX-1, which was used to express the recombinant protein preproHNP-1. The cells were streaked on a Luria−Bertani (LB) plate containing 50 μg/mL of kanamycin as a selective marker and grown overnight at 37 °C. Overnight cultures were prepared by inoculating LB medium containing 50 μg/mL of kanamycin with a single colony. Additionally, the empty vector pET-28a(+) was transformed into *E. coli* BL21 (DE3) competent cells to obtain the strain XPX-2.

Growth rate assessment. The *E. coli* strain XPX-1 was activated overnight by inoculating single colony to 5 mL of LB liquid medium containing 50 μg/mL of kanamycin. The culture was diluted 1:100 diluted with fresh LB liquid medium containing 50 μg/mL of kanamycin to continue growing to an A_{600} between 0.4 and 0.6. Then the culture was split into two parts. One continued to grow while the other was treated with 1mM isopropyl-*β*- D-1-thiogalactopyranoside (IPTG) for the

collection of A_{600} and survival (CFU/mL) samples. Cultures were grown to an A_{600} between 0.4 and 0.6 before treatment, and samples were respectively collected at 90, 180 and 270 min after 1mM IPTG treatment. A_{600} measurements were performed with a Biospec-mini spectrophotometer (Shimadzu, Kyoto, Japan). Fresh LB liquid medium was used as control.

The same overnight culture was used for CFU/mL measurements. One hundred microliters of culture was collected, washed twice with 1 × PBS (pH 7.2), then diluted 1:1000 in 1 × PBS. Ten microliters of each dilution was plated onto Petri dishes containing 20 mL of LB agar, and incubated at 37°C overnight before colony counting. Measurements were taken from at least three different experiments. The mean survival and the standard deviation were calculated across all experiments for each treatment.

Protein extraction, purification of recombinant preproHNP-1 and RecA interacting proteins. The protein purification procedure was based on the method used in our laboratory (1, 2). In brief, after 180 min of IPTG induction, the cells were harvested by centrifugation using a J6-MI centrifuge (Beckman, Brea, CA, USA) at 4,500 ×g for 30 min at 4 °C. The XPX-1 cell pellet was resuspended in PBS and lysed by high-pressure homogenization on an APV 1000 homogenizer (SPX, Charlotte, NC, USA) for three times at 800 bar. The inclusion bodies were collected by centrifugation at 4,500 ×g for 30 min at 4 °C. The pellet was washed with PBS containing 2 M urea for 2 h with stirring. The suspension was centrifuged (4,000 × g, 20 min) to obtain the preproHNP-1 inclusion bodies.

The inclusion bodies were solubilized in denaturing solution (20 mM Tris, 8 M urea, 20 mM DTT, pH 8.5) at a ratio of 1:20 (grams of inclusion body wet weight: mL of volume) and the suspension was shaken gently for 4 h at room temperature. After centrifugation at 12,000 ×g for 15 min, the supernatant was diluted directly into refolding buffer (20 mM Tris,1 mM cystine, 3 mM cysteine, 2 M urea, pH 10.0). After refolding at 4°C overnight, the sample pH was adjusted to 4.5 and the sample was centrifuged at 4,000 ×g for 20 min.

The solubilized protein samples were loaded onto a pre-equilibrated HiTrap Chelating HP (5 mL, GE Healthcare, Chicago, IL, USA) at a flow rate of 10 mL/min in buffer A (20 mM Tris, 2 M NaCl, pH 7.5). The column was then washed with five column volumes (CV) of buffer A followed by a linear gradient to 2% buffer B (20 mM Tris, 2 M NaCl, 0.5 M imidazole, pH 7.5) in 5 CV. The active fraction was eluted with a linear gradient of 2-100% buffer B in 10 CV. Based on SDS-PAGE, the fractions containing preproHNP-1 were pooled together. The purified preproHNP-1 was quantified with BCA according to standard procedures.

To identify the HNP-1 interacting proteins, the XPX-1 and XPX-2 cells containing histidine-tagged preproHNP-1 and vector respectively were grown at 37°C in Luria−Bertani to A_{600} = 0.4 before 1 mM IPTG induction for 1 h. The cell lysates were prepared with PBS as described above. The supernatant was loaded onto a homemade column packed with 20 μL Ni-NTA beads (Qiagen, Hilden, Germany), washed three times with 1 mL of buffer A (20 mM imidazole) and buffer B (40 mM imidazole). The protein targets were eluted with buffer C (250 mM

imidazole) and analyzed by a 12% Tris-Tricine gel followed by Coomassie brilliant blue G-250 staining. The whole lanes were cut into 3-5 pieces for further LC-MS/MS analysis. To identify the RecA interacting proteins, the similar method was used except the Ni-NTA beads replaced with anti-RecA antibody (Abcam, Cambridge, UK) and protein G beads (Thermo, Waltham, MA, USA).

Proteomics analysis. For the global proteomics study, the same amount of total cell lysate (~120 μg) extracted from the XPX-1 and XPX-2 was incubated with 5 mM DTT at 37 °C for 30 min and alkylated with 20 mM IAA in the dark at room temperature for 30 min before run on a 12% Tris-Tricine gel. After the electrophoresis, the gel was stained with Coomassie brilliant blue G-250, and cut into 4 slices based on protein amount and the molecular weight markers. After in-gel digestion by trypsin, the samples were analyzed by LC-MS/MS using a homemade column (75 μm internal diameter ×15 cm long) packed with 3 μm C18 reverse phase beads (Michrom Bioresources, Inc., Auburn, CA, USA), a 60 min gradient was performed on an LTQ-Orbitrap Velos mass spectrometer (Thermo Fisher Scientific, San Jose, CA, USA). MS full scans were performed in an m/z range of 300-1,600 with a resolution of 30,000 at m/z 400. The maximum injection time (MIT) was 150 ms, and the automatic gain control (AGC) was set to 1×10^6. The top 20 intense ions were subjected to Orbitrap for further fragmentation via collision induced dissociation (CID) with the normalized collision energy (NCE) of 35%. For each scan, the AGC was set at 1×10^4 and the MIT was 25 ms. The dynamic exclusion was set 35 s to suppress repeated peak fragmentation.

MS/MS raw files were processed with MaxQuant (version 1.5.6.0, Martinsried, Germany) against a composite target/decoy *E. coli* database (Elias and Gygi, 2007). The target *E. coli* strain K12 complete proteome sequences obtained from UniProt (version from January 2016) plus the 6× his-tagged preproHNP-1 (Uniprot ID: 59665) and common contaminations (3). The parameters for database searching were set as follows: cysteine carbamidomethyl was specified as a fixed modification and oxidation of methionine was set as variable modification. The tolerances of precursor and fragment ions were set at 20 ppm and 0.5 Da, respectively. For digestion, trypsin was set as protease with two missed cleavage permitted. Peptide matches were filtered by a minimum length of seven amino acids. The assigned peptides and proteins were filtered until a false discovery rate lower than 1% was achieved. The quantification of proteins was based on the reporter ion intensity of the identified unique and razor peptides. The global normalization was based on the assumptions that the total intensity of each sample was approximately equal. The value of LFQ was defined as the quantitative value of each protein (4).

The same method was applied for the interacting protein analysis with the enriched protein samples.

Parameter description of the top-down mass spectrometry sample. The same LC-MS/MS platform was used for the full-length mature HNP-1 and its commercially available standard (Sigma, St. Louis, MO, USA). The samples were loaded and eluted with a 60 min liner gradient ramped from 4% to 35% of buffer B (buffer A, 0.1 % acetic acid, 2 % acetonitrile; buffer B, 0.1 % acetic acid, 100 %

acetonitrile) at a flow rate of 0.3 μL/min, as previously described (5). Then eluted HNP-1 molecules were measured in the Orbitrap analyzer at 30000 resolution (at 400 m/z) and a target value of 10^6 ions with a maximum injection time of 150 ms. The 3 most intense ions were isolated within a window of 2 m/z, fragmented under a collision energy of 32-34% and measured in the linear Orbitrap, fragmented and measured in liner ion trap. AGC target was set to 1×10^4 within a maximum injection time of 25 ms. The precursor ion peaks were manually assigned according to the charge state, derived mass and LC retention time.

Preparation of the HNP-1 liposomes. Soybean lecithin was purchased from Taiwei Medicine (Shanghai, China), Cholesterol was purchased from Sinopharm Reagent (Shanghai, China), and chemosynthetic mature HNP-1 and N-[1-(2,3-Dioleoyloxy)propyl]-N,N,N-trimethy-lammonium (DOTAP) were purchased from Sigma (St. Louis, MO, USA). HNP-1 liposomes were prepared with the reverse-evaporation method (6). Briefly, soybean phospholipid, cholesterol and the cationic lipid DOTAP (10:1:1, w/w) were dissolved in dimethyl chloride with ultrasound dissolution. Then HNP-1 dissolved in PBS (pH 6.8) was added. The lipid solutions were mixed in required ratios and the solvent was evaporated under vacuum overnight to leave lipid films. The appropriate amount of PBS were added to hydrate the film with rotation to allow liposome formation. The liposomes were filtrated three times through 0.45 μm sterile membranes (Pall, New York, NY, USA) followed by storage at 4 °C.

Preparation of pneumonic model rats and medicine administration. The clinically isolated bacterial strain MRSA was cultivated in LB medium at 37 °C with continuous shaking (200 rpm) overnight. Then, the concentration of MRSA was determined by the optical density (OD) at 600 nm based on the turbidity of the cell suspension. Subsequently, the bacterial suspension was diluted with LB medium, and a final density of 108 CFU/mL was obtained for animal testing. Male Sprague-Dawley (SD) rats (190–200 g) were provided by Vital River Experimental Animal Technology (Beijing, China). The handlingof the animals and the surgical processes were conducted in accordance with the Guidelines for the Use of Laboratory Animals of Beijing Institute of Radiation Medicine (BIRM) and the animal experiments were approved by the Animal Subject Review Committee. Peripheral blood was collected via tail veins before sacrifice of the animals, then the lung bronchoalveolar lavage fluids (BALFs) were collected and the lung tissues were excised followed by hematoxylin and eosin (H&E) staining.

Bacterial pneumonic rats were prepared by spraying MRSA suspensions into the lung through tracheal routes with a soft long plastic tube as in our previous research (7). The rats were inoculated using tracheal intubation with 0.2 mL of 10^8 CFU/mL MRSA suspensions. After 8 h, the rats were given medications. Twenty-one rats were equally divided into seven groups, including the healthy rats as the controls; the pneumonic rat models treated with saline; the pneumonic rat models treated with blank liposome group; the pneumonic rats treated with 0.1mL 20 μg/mL HNP-1 solutions; the pneumonic rats treated with 0.1 mL 5 μg/mL of HNP-1 liposomes; the

pneumonic rats treated with the 0.1 mL 10 μg/mL of HNP-1 liposomes; the 12 μg/mL sodium penicillin solutions as the quality controls for ensuring the strains resistant to penicillin. All the medicines were sprayed into the rat lungs through the trachea using an intratracheal MicroSprayer Aerosolizer (IA-1B; PennCentury Inc., Wyndmoor, PA, USA) once daily for three days without anaesthesia.

Scanning electron microscopy analysis. Samples were prepared as previously described in the literature (8, 9). Briefly, approximately 5×10^8 cells were collected and washed in phosphate buffer (0.1 M, pH 7.2). The cells were fixed overnight at 4°C in 2.5% glutaraldehyde, washed with same phosphate buffer for three times. Then the fixed cells were postfixed with 0.1 M phosphate buffer containing 2% osmium tetroxide (OsO_4) (pH 7.2) for 20 min. After dehydration with a stepwise series of ethanol solution with increasing concentration (50%、70%、85% and 95%) for 20 min respectively, the cells were coated with platinum by standard procedures. Samples were examined using an EVO LS10 scanning electron microscope (ZEISS, Jena, Germany), and images were processed by SmartSEM (ZEISS, Jena, Germany).

Analysis of DNA fragmentation by TUNEL. The DNA fragments were labeled using the Apo-Direct Kit (BD Bioscience, Oakland, CA, USA), which employs FITC-conjugated deoxyuridine triphosphate (FITC-dUTP) for staining and propidium iodide as a counterstain to characterize the dead cells. Sample preparation methods were performed according to the previous literature (10). Approximately 10^6 cells were collected，washed once and resuspended in 500 μL cold PBS (pH 7.2). 1 mL of 4% paraformaldehyde was added to each sample for 30 min on ice to fix

cells. Cells were collected by centrifugation at 8,000 rpm for 5 min, washed once and resuspended in PBS. Then 1 mL of ice cold 70% ethanol was added to each sample to store at -20 °C overnight to increase cell permeability.

For staining, samples were spun down at 4 °C 8,000 rpm for 5 min in a refrigerated microcentrifuge to remove the ethanol. Samples were washed twice in 1 mL wash buffer (kit component), and removed the supernatant after the second wash. The cells were resuspended with 50 µL DNA labeling solution (kit components), and then incubated for 60 min at 37°C. To stop the staining reaction, 1 mL of rinse buffer (kit components) was added followed by centrifugation at 8,000 rpm for 5 min. The rinsing step was repeated, the cell pellet was resuspended in 500 µL of propidium iodide staining buffer, and the cells were incubated in the dark for 30 min at room temperature. Next, analyzed the cells in staining buffer by flow cytometry (BD Bioscience, Oakland, CA, USA).

Analysis of phosphatidylserine exposure by flow cytometry. At each time point, approximately 10^6 cells were collected, washed once and resuspended in 1 mL sterile distilled water. Samples were spun down at 3,000 g for 5 min in a refrigerated microcentrifuge, then resuspended in 100 µL of 1 × annexin- binding buffer. Five microliters of FITC-conjugated annexin V and 5 µL of propidium iodide (DOJINDO, Kumamoto-ken, Japan) were added to each sample followed by incubation in the dark for 15 min at room temperature. Samples were diluted by adding 400 µL 1 × annexin-binding buffer. Then detected phosphatidylserine exposure by flow cytometry.

Western blot analysis. Equal amounts of eluted proteins (HNP-1 and vector) were separated by 12% Tris-Tricine gel, transferred to 0.2 μm nitrocellulose blotting membranes (Merck Millipore, Darmstadt, Germany) blocked with 5% non-fat milk powder in TBS-T (20mM Tris-HCl, 150mM NaCl and 0.05% Tween-20). Primary antibodies of preproHNP-1 were purchased from Santa Cruz Biotechnology (Dallas, TX, USA) and primary antibodies against RecA were purchased from Abcam (Cambridge, UK). The primary antibodies were detected using horseradish peroxidase (HRP)-conjugated secondary antibodies (Abcam, Cambridge, UK) and a chemiluminescent detection reagent (Thermo, Waltham, MA, USA).

Immunofluorescence staining. Immunofluorescence (IF) staining was performed using an anti-Annexin V rabbit polyclonal antibody (Abcam, Cambridge, UK) and an anti-Staphylococcus aureus mouse monoclonal antibody (Abcam, Cambridge, UK) to determine the bacterial apoptosis. The secondary antibodies used were Alexa Fluor 594 goat anti-rabbit IgG (Invitrogen, Carlsbad, CA, USA) and Alexa Fluor 488 goat anti-mouse IgG (Invitrogen, Carlsbad, CA, USA). IF staining was performed according to standard procedures. Briefly, the frozen slices were incubated with goat serum for 40 min at 37 °C after washing with phosphate buffered saline (PBS). Subsequently, slices were stained with 1:100 dilution of anti-Annexin V and anti-S. aureus antibodies overnight at 4 °C. Secondary antibodies were added for staining at 37 °C for 40 min. The slices were then analyzed with a Zeiss LSM 880 confocal laser scanning microscope (Zeiss, Oberkochen, Germany).

Analysis of gene expression by real-time RT-PCR. mRNA from RecA and LexA was measured by real-time PCR (RT-PCR). XPX-1 cells harvested 3 h after induction and the total RNA was extracted with the TRIzol® Reagent (Thermo Fisher Scientific, Waltham, MA, USA) according to the manufacturer's instruction. Reversed transcription was performed with the ReverTra Ace qPCR RT Kit (TOYOBO, Osaka, Japan). The primer sequence of RecA and LexA was as follows: RecA (forward 5'-GAAATCTTTCGGTAAAGGTGCC-3', reverse 5'-CGCCTAATGCTTGAGCATATTC-3'); LexA (forward 5'-GCACGTAGCGTATCAACAGCT-3 ' , reverse 5 ' -AGAGAATTTAGCATATGGTC-3'). The relative expression level of RecA and LexA was normalized to 16s by using the $2^{-\triangle\triangle}$CT method(11).

Measurement of IL-6 and IFN-γ. Serum was separated by centrifugation of the blood samples from seven groups rats Interleukin-6 (IL-6) and Interferon-γ (IFN-γ) in the rats were measured with the commercial ELISA assay kits (Neobioscience Technology Co., Ltd., Shenzhen, China). The absorbance of IL-6 and IFN-γ was measured at 450 nm, and quantification was conducted according to the manufacturers' instructions. All procedures were performed in accordance with the manufacturer's instructions.

References:

1. Zhao M, Cai M, Wu F, Zhang Y, Xiong Z, Xu P. 2016. Recombinant expression, refolding, purification and characterization of Pseudomonas aeruginosa protease IV in Escherichia coli. Protein Expr Purif 126:69-76. https://doi.org/10.1016/j.pep.2016.05.019
2. Zhao M, Wu F, Xu P. 2015. Development of a rapid high-efficiency scalable process for acetylated Sus scrofa cationic trypsin production from Escherichia coli inclusion bodies. Protein Expr Purif 116:120-6. https://doi.org/10.1016/j.pep.2015.08.025

3. Ping L, Zhang H, Zhai L, Dammer EB, Duong DM, Li N, Yan Z, Wu J, Xu P. 2013. Quantitative proteomics reveals significant changes in cell shape and an energy shift after IPTG induction via an optimized SILAC approach for Escherichia coli. J Proteome Res 12:5978-88. https://doi.org/10.1021/pr400775w
4. Ju¨ rgen Cox MYH, Christian A. Luber, Igor Paron, Nagarjuna Nagaraj, Matthias Mann. 2014. Accurate proteome-wide label-free quantification by delayed normalization and maximal peptide ratio extraction. Molecular & Cellular Proteomics 13:2513-2526. https://doi.org/10.1074/mcp.M113.031591
5. Ping Xu JP. 2008. Characterization of polyubiquitin chain structure by middle-down mass spectrometry. Analytical Chemistry 80:3438-44. https://doi.org/10.1021/ac800016w
6. Kim BK, Hwang GB, Seu YB, Choi JS, Jin KS, Doh KO. 2015. DOTAP/DOPE ratio and cell type determine transfection efficiency with DOTAP-liposomes. Biochim Biophys Acta 1848:1996-2001. https://doi.org/10.1016/j.bbamem.2015.06.020
7. Li M, Zhang T, Zhu L, Wang R, Jin Y. 2017. Liposomal andrographolide dry powder inhalers for treatment of bacterial pneumonia via anti-inflammatory pathway. Int J Pharm 528:163-171. https://doi.org/10.1016/j.ijpharm.2017.06.005
8. Ping X, Takahashi Y, Seino A, Iwai Y, Omura S. 2004. Streptomyces scabrisporus sp. nov. Int J Syst Evol Microbiol 54:577-581. https://doi.org/10.1099/ijs.0.02692-0
9. Dewachter L, Verstraeten N, Monteyne D, Kint CI, Versees W, Perez-Morga D, Michiels J, Fauvart M. 2015. A Single-Amino-Acid Substitution in Obg Activates a New Programmed Cell Death Pathway in Escherichia coli. mBio 6:e01935-15. https://doi.org/10.1128/mBio.01935-15
10. Dwyer DJ, Camacho DM, Kohanski MA, Callura JM, Collins JJ. 2012. Antibiotic-induced bacterial cell death exhibits physiological and biochemical hallmarks of apoptosis. Mol Cell 46:561-72. https://doi.org/10.1016/j.molcel.2012.04.027
11. Schmittgen TD, Livak KJ. 2008. Analyzing real-time PCR data by the comparative C(T) method. Nat Protoc 3:1101-8. https://doi.org/10.1038/nprot.2008.73

TABLE S1 Strains used in this study.

Strain	Host	Genotype	Description	Source or reference
XPX-1	BL21(DE3)	*F- ompT hsdSB (rBmB-) gal dcm (DE3)*	*E. coli* containing pET-28a (+)- preproHNP-1	This study
XPX-2	BL21(DE3)	*F- ompT hsdSB (rBmB-) gal dcm (DE3)*	*E. coli* containing pET-28a (+)	This study

TABLE S2 MS information of the preproHNP-1 and HNP-1.

Protein	Unique peptides	MW [kDa]	Sequence coverage [%]	Intensity	MS/MS count
PreproHNP-1	5	14.8	53.60	27708000	14
HNP-1	2	3.4	60.00	1208400	2

TABLE S3 The list of differentially expressed proteins.

The list of upregulated proteins.

Majority protein ID	Gene name	Intensity (XPX-1+2)	Log2 ratio (XPX-1/2)	Significance B
P0ABP8	deoD	3.57E+06	5.4	0.0013
P0A898	ybeY	2.10E+07	5.39	0.0002
P0ABA0	atpF	1.79E+07	5.23	0.0003
P0ADW3	yhcB	1.97E+06	5.17	0.002
P0A7J3	rplJ	5.33E+06	5.08	0.0024
P39396	yjiY	2.09E+07	5.04	0.0005
P0AFD4	nuoH	1.60E+08	4.9	0
P0ADT8	ygiM	4.60E+06	4.85	0.0038
P59665	HNP1	1.13E+07	4.71	0.005
P0AGA2	secY	1.23E+06	4.55	0.0067
P69786	ptsG	2.30E+07	4.27	0.0035
P0ABC3	hflC	8.05E+06	4.1	0.0146
P00573	T7	2.76E+07	4.05	0.0057
P42596	rlmG	9.12E+06	3.99	0.0177
P36680	zapD	2.53E+06	3.96	0.0186
P0A7A9	ppa	1.04E+07	3.94	0.0191
P0A9P6	deaD	1.51E+07	3.85	0.0086

P0ABK2	cydB	1.19E+07	3.77	0.025
P21513	rne	1.21E+07	3.77	0.0251
P0AG27	yibN	7.19E+08	3.76	0.0011
P0AE78	corC	3.31E+06	3.73	0.0265
P14175	proV	1.39E+07	3.68	0.0287
P0A7K2	rplL	2.44E+07	3.59	0.0146
P76445	lpxT	6.07E+06	3.56	0.0346
P0ABZ4	kdsC	1.52E+07	3.51	0.017
P0A6T1	pgi	2.42E+07	3.5	0.0175
P27434	rodZ	1.37E+06	3.49	0.0382
P0A7T7	rpsR	1.36E+07	3.46	0.0401
P06710	dnaX	6.94E+06	3.4	0.0438
P25552	gppA	1.60E+09	3.36	0.0034
P77173	zipA	2.01E+06	3.36	0.0461
P07118	valS	3.00E+07	3.36	0.0226
P75838	ycaO	7.90E+06	3.35	0.0471
P43329	hrpA	1.31E+07	3.34	0.0476
P11454	entF	9.96E+07	3.31	0.0039
P0A6X3	hfq	7.16E+07	3.29	0.0259
P60651	speB	2.94E+07	3.28	0.0264
P75876	rlmI	1.94E+08	3.26	0.0044

P23930	lnt	3.84E+07	3.24	0.0284
P00452	nrdA	2.07E+07	3.23	0.0285
P0A7M2	rpmB	4.11E+07	3.23	0.0286
P0C0L7	proP	1.78E+07	3.19	0.0307
P75691	yahK	3.54E+07	3.16	0.0326
P0A8K1	psd	1.13E+08	3.14	0.0062
P12758	udp	5.68E+08	3.12	0.0065
P0A8W8	yfbU	3.18E+07	3.1	0.0359
P0AF20	nagC	2.36E+07	3.08	0.0375
P0A7X3	rpsI	6.12E+07	3.05	0.0394
P0AG93	secF	1.46E+09	3.03	0.0081
P0ADY7	rplP	1.39E+08	2.98	0.0092
P09155	rnd	9.03E+07	2.97	0.0097
P07821	fhuC	2.29E+08	2.94	0.0104
P0CB39	eptC	2.66E+07	2.91	0.0499
P0A6R3	fis	2.73E+08	2.77	0.0155
P0ADZ7	yajC	3.22E+08	2.66	0.02
P11557	damX	1.34E+08	2.6	0.0234
P60624	rplX	1.99E+08	2.54	0.0265
P0ABF1	pcnB	1.26E+08	2.49	0.0298
P30845	eptA	9.93E+07	2.47	0.0306

P0CG19	rph	8.04E+07	2.35	0.0395
P0A7L3	rplT	1.44E+08	2.3	0.0443

The list of downregulated proteins.

Majority protein ID	Gene name	Intensity (XPX-1+2)	Log2 ratio (XPX-1/2)	Significance B
P77318	ydeN	1.19E+08	-11.55	0
P0A853	tnaA	8.11E+07	-10.37	0
P77269	yphF	2.41E+07	-9.22	0
P0A9P9	idnO	1.03E+08	-7.96	0
P39346	idnD	8.89E+07	-6.56	0
P25553	aldA	7.81E+07	-6.45	0
P06720	melA	1.07E+09	-6.38	0
P21177	fadB	1.21E+07	-6.2	0.0005
P19926	agp	1.82E+08	-6.14	0
P37188	gatB	3.01E+07	-6.07	0
P76128	ddpA	1.30E+06	-6.06	0.0006
P76585	yphG	1.28E+08	-6.02	0
P77252	ykgE	1.33E+07	-5.98	0.0008
P0A9S3	gatD	1.85E+08	-5.78	0
P0A9G6	aceA	1.13E+08	-5.66	0
P69828	gatA	2.05E+08	-5.59	0

P77748	ydiJ	1.37E+08	-5.51	0
P0ADE6	ygaU	1.15E+07	-5.48	0.002
P0ABQ2	garR	2.08E+08	-5.32	0
P09394	glpQ	1.61E+07	-5.21	0.0002
Q46863	ygiS	3.26E+07	-5.12	0.0002
P27550	acs	1.27E+08	-5.06	0
P0AG76	sbcD	2.02E+07	-5.04	0.0003
P09551	argT	4.83E+07	-4.95	0.0004
P0A7C6	pepE	2.22E+07	-4.93	0.0004
P0C8J6	gatY	5.70E+07	-4.82	0.0006
P0AFH8	osmY	4.78E+07	-4.8	0.0006
P33232	lldD	2.60E+08	-4.76	0
P46888	uspC	3.85E+06	-4.73	0.0077
P25748	galS	1.39E+07	-4.72	0.0077
P0A8G6	wrbA	1.61E+08	-4.7	0
P77739	yniA	2.01E+07	-4.68	0.0008
P0C8J8	gatZ	2.67E+08	-4.61	0
P76149	sad	5.45E+07	-4.54	0.0011
P37903	uspF	6.05E+07	-4.53	0.0011
P0A8D6	ymdB	5.27E+06	-4.44	0.0121
P76216	astB	6.15E+05	-4.35	0.0141

P76052	abgB	1.52E+06	-4.33	0.0146
P37902	gltI	2.62E+08	-4.15	0
P0A6F3	glpK	1.73E+08	-3.98	0
P77581	astC	2.80E+07	-3.97	0.0042
P23538	ppsA	2.34E+08	-3.97	0
P28629	adiA	1.36E+07	-3.95	0.0257
P10907	ugpC	1.93E+06	-3.93	0.0264
P78067	ynjE	1.93E+07	-3.87	0.0052
P0AAT9	ybeL	1.11E+07	-3.83	0.0304
P00805	ansB	4.72E+06	-3.78	0.0323
P0A761	nanE	2.36E+06	-3.78	0.0325
P0AC38	aspA	3.57E+08	-3.71	0.0001
P0AFW8	rof	1.00E+07	-3.67	0.0379
P0A9A2	ftnB	1.28E+07	-3.66	0.0381
P02925	rbsB	3.87E+07	-3.66	0.0081
P0C0L2	osmC	1.68E+07	-3.63	0.0086
P76004	ycgM	1.07E+07	-3.63	0.0401
P33570	tktB	3.77E+07	-3.62	0.0088
P42593	fadH	1.50E+06	-3.58	0.0428
P76193	ynhG	1.20E+07	-3.46	0.0498
P0A867	talA	5.70E+07	-3.43	0.0129

P02943	lamB	2.08E+08	-3.42	0.0004
P23847	dppA	8.37E+07	-3.42	0.0004
P0AAB8	uspD	2.28E+07	-3.36	0.0147
P0AC59	grxB	1.26E+08	-3.19	0.001
P17846	cysI	2.11E+07	-3.16	0.0215
P16700	cysP	3.65E+07	-3.13	0.0228
P00722	lacZ	9.19E+08	-3.1	0.0014
P08331	cpdB	2.21E+08	-2.99	0.0021
P0AET8	hdhA	3.71E+07	-2.98	0.0298
P07003	poxB	1.57E+07	-2.98	0.03
P56262	ysgA	2.80E+07	-2.95	0.0314
P0AEQ3	glnH	2.89E+08	-2.71	0.0053
P0A7B1	ppk	2.14E+07	-2.69	0.0486
P69776	lpp	2.55E+07	-2.69	0.0487
P76558	maeB	1.97E+08	-2.69	0.0056
Q46857	dkgA	2.78E+07	-2.69	0.049
P75797	gsiB	3.44E+07	-2.67	0.05
P09424	mtlD	1.26E+08	-2.57	0.0081
P05042	fumC	1.46E+08	-2.55	0.0088
P23843	oppA	8.63E+08	-2.54	0.0089
P0A7Z0	rpiA	1.31E+08	-2.5	0.0102

P0A6J5	dadA	8.56E+07	-2.46	0.0115
P39177	uspG	2.06E+08	-2.43	0.0124
P06999	pfkB	2.04E+08	-2.26	0.0201
P61889	mdh	1.67E+09	-2.24	0.0217
P0AC33	fumA	3.36E+08	-2.2	0.0236
P09546	putA	1.28E+09	-2.15	0.0273
P0AEX9	malE	9.20E+08	-2.11	0.03
P22525	ycbB	9.28E+07	-2.08	0.0331
P0ABH7	gltA	6.27E+08	-2.06	0.0348

TABLE S4 The list of HNP-1 interacting proteins.

Majority protein ID	Gene name	Exp1. LOG10 ratio (XPX-1/2)	Exp2. LOG10 ratio (XPX-1/2)
P59665	DEFA1	6.97	7.12
P1A7G6	recA	6.64	6.81
P77814	ydgA	6.56	6.53
P62621	ispG	6.55	6.45
P1A8V2	rpoB	6.54	6.45
P36979	rlmN	6.51	6.27
P12931	tolC	6.44	6.57
P1A7X3	rpsI	6.44	5.45
P1A6H1	clpX	6.43	6.54
P32132	typA	6.41	6.15
P1AGA2	secY	6.4	6.38
P75821	ybjS	6.4	6.33
P11957	alaS	6.35	6.11
P17114	sdhB	6.29	6.07
P16971	fhuA	6.28	6.21
P31554	lptD	6.28	6.17
P33941	mqo	6.23	6.13
P46889	ftsK	6.2	5.62
P1ACB1	dnaB	6.18	6.2

P36683	acnB	6.17	6.08
P27434	rodZ	6.16	6.09
P24252	ybgA	6.16	4.58
P77536	ykgF	6.15	5.75
P1A9Q7	adhE	6.15	5.79
P75829	ybjX	6.15	6.05
P1ACE1	hybC	6.13	6.14
P13119	metH	6.12	5.9
P1AFX4	rsd	6.11	5.89
P36672	treB	6.11	6.52
P18516	dacC	6.11	6.08
P22523	mukB	6.08	6.05
P17124	ushA	6.07	5.92
P1AEE1	dcrB	6.07	6.06
P77173	zipA	6.06	6.38
P1C158	ibpB	6.06	5.61
P1A698	uvrA	6.04	6.12
P1C8J8	gatZ	6.03	6.11
P12918	mrcA	6	6.05
P1A9M8	pta	5.95	5.96
P1A6Y5	hslO	5.94	5.33

P76658	hldE	5.91	5.71
P61785	lepA	5.9	5.86
P1A915	slyB	5.9	6.11
P1A9K3	ybeZ	5.89	5.89
P33136	mdoG	5.88	5.73
P17118	valS	5.87	5.84
P19127	hemX	5.87	5.5
P77529	ydjN	5.86	5.55
P61752	msbA	5.86	5.65
P77775	yfcH	5.86	5.83
P77757	arnC	5.86	5.96
P1ABD8	accB	5.85	5.62
P1AG93	secF	5.85	5.88
P67911	hldD	5.84	5.6
P69425	tatB	5.84	5.37
P1A9S3	gatD	5.84	5.8
P17443	murG	5.84	5.65
P1AG24	spoT	5.83	5.93
P1A6K6	deoB	5.83	5.9
P1C1S1	mscS	5.79	5.5
P18957	hsdM	5.78	5.33

P61716	lipA	5.76	5.66
P11551	mtlA	5.75	5.33
P1A887	ubiE	5.75	6.55
P37665	yiaD	5.75	5.62
P1ABH7	gltA	5.75	5.81
P1A959	alaA	5.75	5.63
P1AER8	gltS	5.74	5.75
P75828	ybjD	5.73	5.45
P17315	cirA	5.73	5.42
P29131	ftsN	5.73	5.64
P76445	lpxT	5.73	5.47
P1AA16	ompR	5.72	5.47
P1AG11	wzzE	5.72	5.64
P1ADA5	yajG	5.71	5.53
P22333	add	5.68	5.71
P11371	gdhA	5.66	5.81
P1AB24	efeO	5.66	5.47
P1AB67	pntB	5.66	5.64
P1AFF6	nusA	5.66	5.62
P1AES4	gyrA	5.63	5.62
P18395	sppA	5.62	5.77

P1A9L3	fklB	5.62	5.77
P69874	potA	5.62	5.64
P1C1V1	degP	5.6	5.68
P1A927	tsx	5.59	5.46
P77444	sufS	5.59	5.62
P77488	dxs	5.58	5.59
P1ADK1	yiaF	5.58	6.36
P13136	fecA	5.57	5.25
P13135	glpD	5.56	5.42
P77433	ykgG	5.55	5.16
P19934	tolA	5.55	5.5
P1A799	pgk	5.53	5.33
P63386	mlaF	5.53	5.16
P27313	emrA	5.53	5.5
P27836	wecG	5.52	5.27
P28249	asmA	5.52	5.6
P1ACB7	hemY	5.52	5.38
P1ACB4	hemG	5.52	5.64
P17952	murC	5.52	5.71
P1A9W3	ettA	5.51	5.37
P69786	ptsG	5.5	5.56

P1ACL2	exuR	5.5	4.72
P1A7L3	rplT	5.5	5.91
P1AFD6	nuoI	5.46	5.92
P1AFD4	nuoH	5.46	5.4
P17846	cysI	5.46	5.38
P1ADA3	nlpD	5.44	5.25
P1A7B8	hslV	5.43	6.08
P12919	mrcB	5.43	5.23
P1AC12	bamD	5.4	4.76
P11441	lpxB	5.4	5.27
P11961	glyS	5.4	4.95
P1AAD6	sdaC	5.38	6.22
P69831	gatC	5.37	5.2
P1A9V1	lptB	5.37	5.8
P1A8K1	psd	5.36	5.41
P37113	norR	5.34	5.33
P76268	kdgR	5.34	5.79
P1A8N5	lysU	5.34	4.71
P31433	yicH	5.34	5.16
P1A8R7	ycjF	5.33	5.35
P22524	mukE	5.33	4.97

P1A935	mltA	5.33	5.54
P1AD68	ftsI	5.31	5.08
P1AAF6	artP	5.31	5.44
P32176	fdoG	5.31	4.9
P1AES6	gyrB	5.31	6.26
P1AEU1	hisJ	5.31	5.23
P76578	yfhM	5.3	4.79
P1AFC3	nuoA	5.29	5.53
P1A749	murA	5.29	5.3
P25535	ubiI	5.29	4.88
P17993	ubiG	5.28	5.4
P1A9F3	cysB	5.26	4.93
P1ADP2	yigI	5.26	5.67
P75728	ubiF	5.25	4.62
P11959	metG	5.24	4.72
P1AFF2	nupC	5.21	5.11
P67187	rsmI	5.2	5.34
P1AE82	cpxA	5.19	5.24
P1A722	lpxA	5.19	5.11
P14294	topB	5.17	5.37
P21599	pykA	5.16	4.92

P16612	topA	5.14	5.92
P16616	era	5.14	5.06
P76473	arnT	5.14	4.99
P1AD65	mrdA	5.13	5.38
P12359	rpsG	5.13	5.34
P77395	ybbN	5.13	4.94
P1ABV6	tolR	5.13	4.75
P76472	arnD	5.12	5.06
P21499	rnr	5.09	5.54
P31744	sdaB	5.08	4.71
P31547	metI	5.07	4.89
P46837	yhgF	5.07	4.91
P1ADC6	lptG	5.07	5.36
P1AFG1	nusG	5.01	5.38
P1ABU7	exbB	5	5.63
P1ABI4	corA	5	5.47
P19323	nagE	5	5.06
P1ADZ7	yajC	4.98	5.77
P14176	proW	4.98	5.06
P16711	dnaX	4.98	4.9
P37151	purU	4.98	5.09

P1A6P5	der	4.97	4.67
P64596	yraP	4.97	5.59
P1AAJ8	hybA	4.96	4.73
P14993	recD	4.95	5.06
P62517	mdoH	4.92	5.11
P1AEC3	arcB	4.91	4.79
Q46925	csdA	4.9	5
P1AAT4	ybdG	4.87	4.91
P1ADY7	rplP	4.87	6.23
P77718	thiI	4.85	5.28
P75831	macA	4.83	4.96
P1A6J5	dadA	4.83	5.76
P1AGB6	rpoE	4.83	5.44
P11813	lepB	4.64	5.5
P25888	rhlE	4.64	5.08
P76372	wzzB	4.49	5.88

TABLE S5 The identification of RecA protein by MS.

	Protein	Unique peptides	Sequence coverage [%]	Intensity	MS/MS count
TEST1	RecA	14	47.30	9333111	21
TEST2	RecA	15	50.40	13341111	24

TABLE S6 MS information of proteins which molecular weight less than 10 kDa.

Protein	Unique peptides	MW [kDa]	Sequence coverage [%]	Intensity	MS/MS count
rpmB	1	9	12.8	247140	1
rpmJ	1	4.4	21.1	583940	1
rpmC	1	7.3	22.2	121300	1
ECBD_1968	1	8.3	19.9	146440	1
Mature HNP-1	2	3.4	60	1208400	2

A.

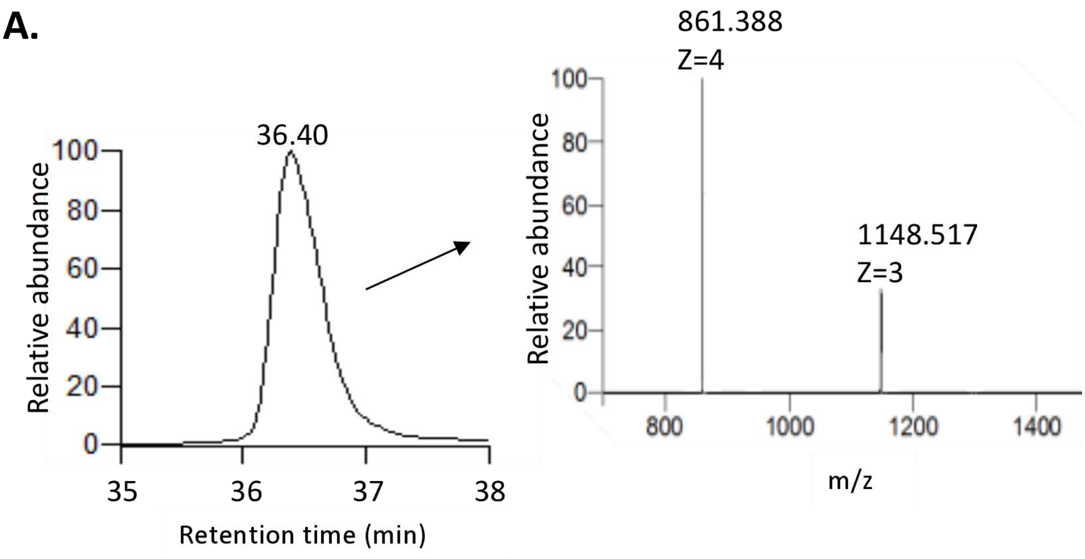

Standard HNP-1

B.

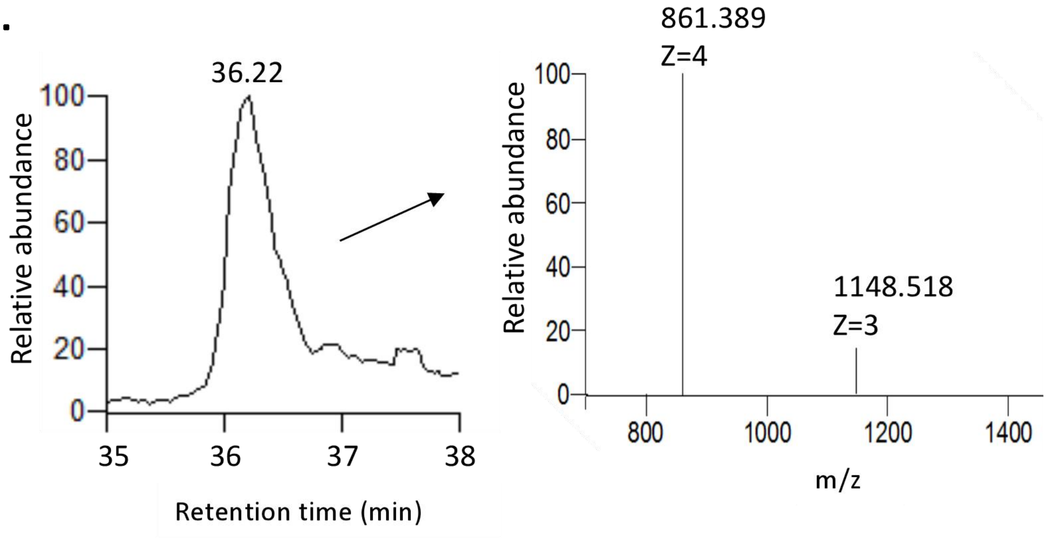

HNP-1

FIGURE S1. (A) Top-down analysis for the standard HNP-1 by LTQ-Orbitrap-Velos. The inset shows the MS spectrum and monoisotopic m/z values of the detected HNP-1 protein. **(B)** Top-down analysis of the purified HNP-1 by LTQ-Orbitrap-Velos. The inset shows the MS spectrum and monoisotopic m/z values of the detected HNP-1 protein.

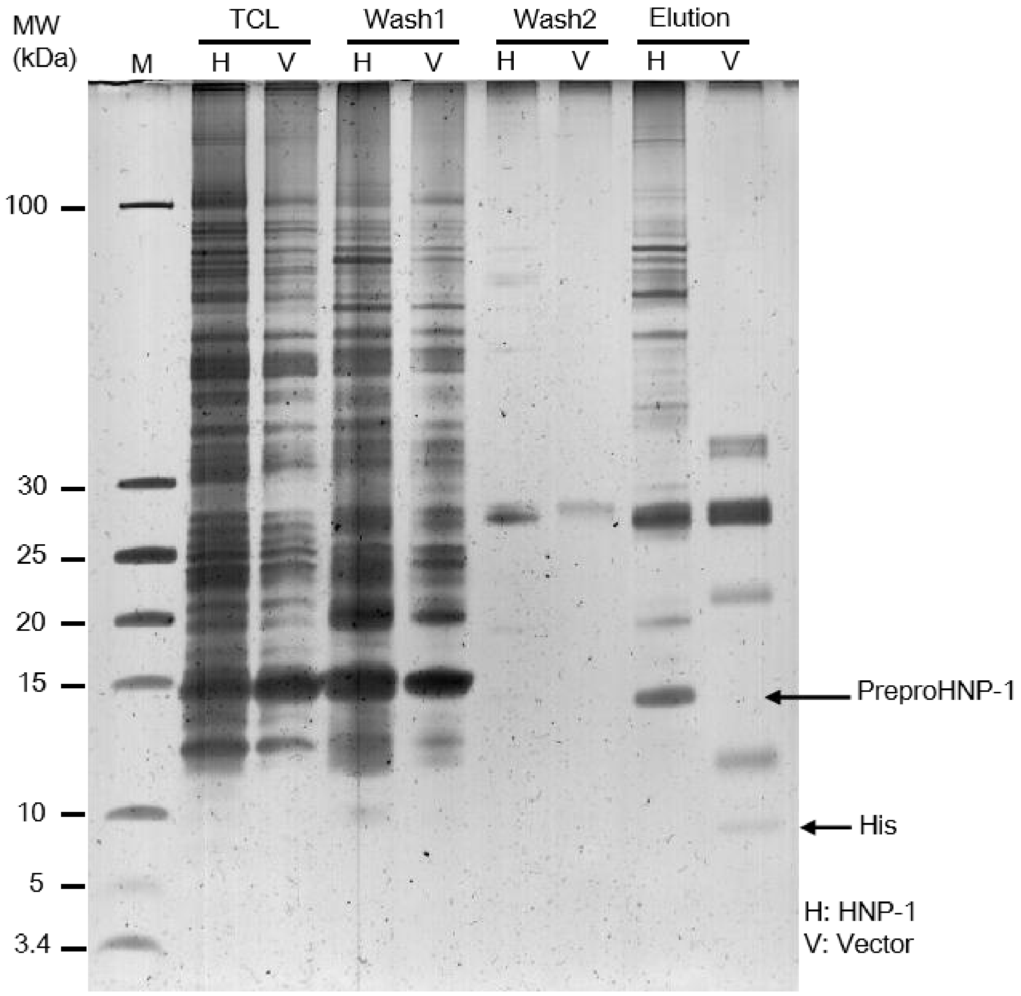

FIGURE S2. Tris-Tricine gel analysis of the HNP-1 interacting proteins. Cell lysates containing the HNP-1 sequence and vector sequence were prepared with PBS, loaded onto a homemade column packed with 20-μL Ni-NTA beads, washed three times with 1 mL of buffer 1 (20 mM imidazole) and wash buffer 2 (40 mM imidazole). Then the protein targets were eluted with elution buffer (250 mM imidazole), and all samples were analyzed by a 12% Tris-Tricine gel followed by silver staining.

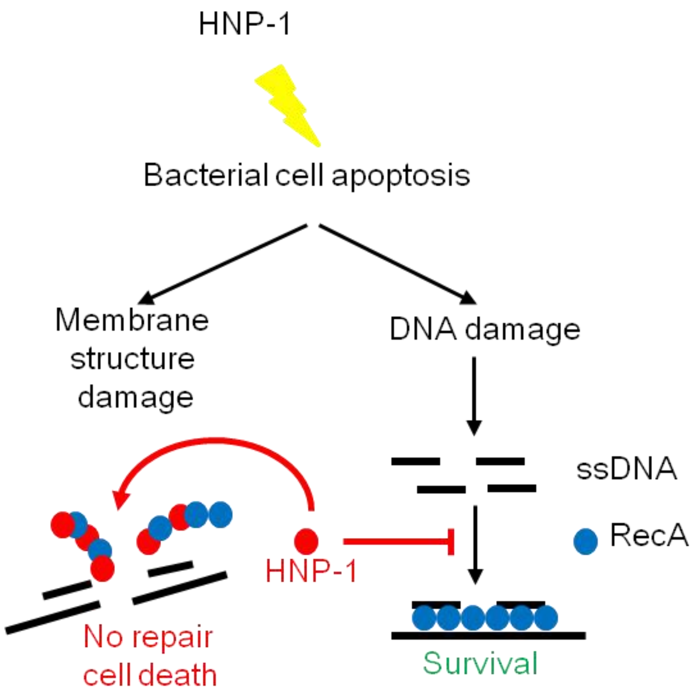

FIGURE S3. Hypothesized pathways of bacterial apoptosis caused by HNP-1. HNP-1 interferes with the binding of RecA and ssDNA, thereby disrupting the DNA damage repair response during bacterial apoptosis.

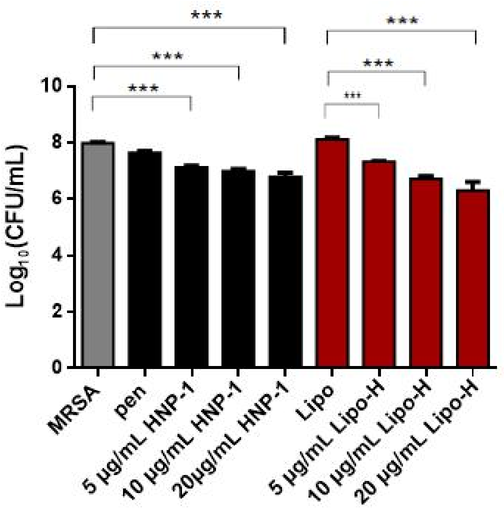

FIGURE S4. The antibacterial activity of the treatments against MSRA *in vitro.* The *in vitro* anti-MSRA effect of penicillin, HNP-1, and liposomal HNP-1 with gradient concentrations. Data were analyzed using a two-tailed Student's *t* test and are plotted as the mean ± SD for each condition.

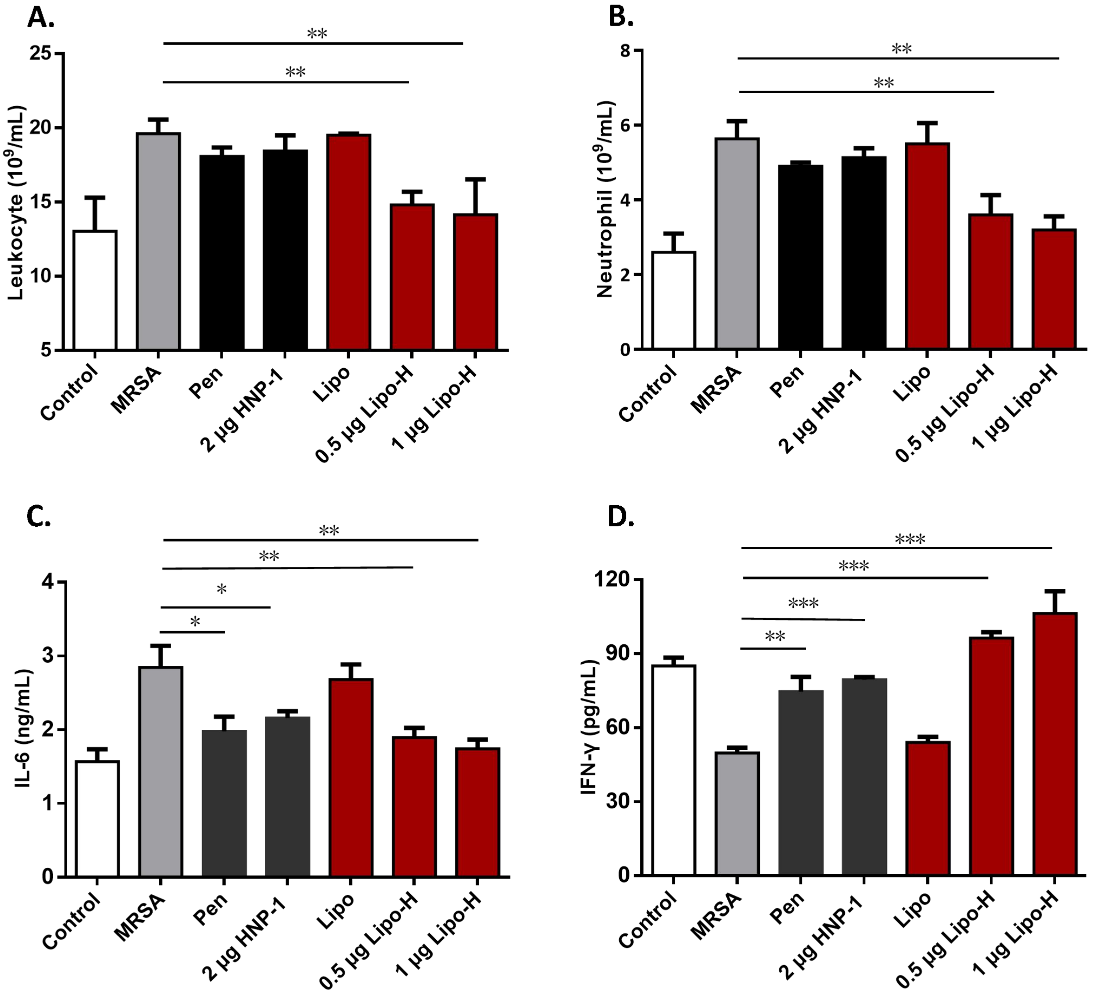

FIGURE S5. Effects of the medicines on the innate immunity. Twelve hours after the final administration of the treatments, tail vein blood (20 μL) was collected. The number of leukocytes (**A**) and neutrophils (**B**) were counted an automated hematology analyzer. Interleukin-6 (IL-6) (**C**) and interferon-γ (IFN-γ) (**D**) levels were measured with commercial ELISA assay kits. Data were analyzed using a two-tailed Student's *t* test and are plotted as the mean ± SD for each condition. **P* < 0.05, ***P* < 0.01, and ****P* < 0.001.

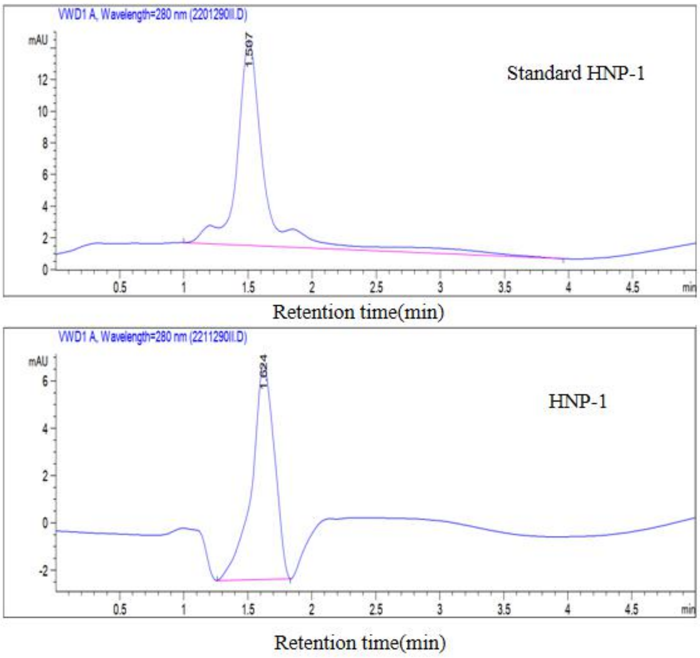

	Retentin time（min）	Area	Concentration（μg/mL）
Standard HNP-1	1.507	201.50117	1.00
HNP-1	1.624	112.82637	0.56

FIGURE S6. Quantitative HPLC analysis of HNP-1. The same HPLC platform was used to examine mature HNP-1 after ultrafiltration and the commercially available HNP-1 standard.
